# Development of a tissue-specific ribosome profiling approach in *Drosophila* enables genome-wide evaluation of translational adaptations

**DOI:** 10.1371/journal.pgen.1007117

**Published:** 2017-12-01

**Authors:** Xun Chen, Dion Dickman

**Affiliations:** 1 Department of Neurobiology, University of Southern California, Los Angeles, California, United States of America; 2 USC Neuroscience Graduate Program, University of Southern California, Los Angeles, California, United States of America; New York University, UNITED STATES

## Abstract

Recent advances in next-generation sequencing approaches have revolutionized our understanding of transcriptional expression in diverse systems. However, measurements of transcription do not necessarily reflect gene translation, the process of ultimate importance in understanding cellular function. To circumvent this limitation, biochemical tagging of ribosome subunits to isolate ribosome-associated mRNA has been developed. However, this approach, called TRAP, lacks quantitative resolution compared to a superior technology, ribosome profiling. Here, we report the development of an optimized ribosome profiling approach in *Drosophila*. We first demonstrate successful ribosome profiling from a specific tissue, larval muscle, with enhanced resolution compared to conventional TRAP approaches. We next validate the ability of this technology to define genome-wide translational regulation. This technology is leveraged to test the relative contributions of transcriptional and translational mechanisms in the postsynaptic muscle that orchestrate the retrograde control of presynaptic function at the neuromuscular junction. Surprisingly, we find no evidence that significant changes in the transcription or translation of specific genes are necessary to enable retrograde homeostatic signaling, implying that post-translational mechanisms ultimately gate instructive retrograde communication. Finally, we show that a global increase in translation induces adaptive responses in both transcription and translation of protein chaperones and degradation factors to promote cellular proteostasis. Together, this development and validation of tissue-specific ribosome profiling enables sensitive and specific analysis of translation in *Drosophila*.

## Author summary

Recent advances in next-generation sequencing approaches have revolutionized our understanding of transcriptional expression in diverse systems. However, transcriptional expression alone does not necessarily report gene translation, the process of ultimate importance in understanding cellular function. Ribosome profiling is a powerful approach to quantify the number of ribosomes associated with each mRNA to determine rates of gene translation. However, ribosome profiling requires large quantities of starting material, limiting progress in developing tissue-specific approaches. Here, we have developed the first tissue-specific ribosome profiling system in *Drosophila* to reveal genome-wide changes in translation. We first demonstrate successful ribosome profiling from muscle cells that exhibit superior resolution compared to other translational profiling methods. We then use transcriptional and ribosome profiling to define whether transcriptional or translational mechanisms are necessary for synaptic signaling at the neuromuscular junction. Finally, we utilize ribosome profiling to reveal adaptive changes in cellular translation following cellular stress to muscle tissue. Together, this now enables the power of *Drosophila* genetics to be leveraged with ribosome profiling in specific tissues.

## Introduction

Recent advances in next-generation sequencing such as RNA-seq have revolutionized the measurement and quantification of genome-wide changes in transcriptional expression, without pre-existing knowledge of gene identity, at unprecedented resolution [1, 2]. In addition, biochemical tagging of ribosomes has emerged as a powerful way to provide insight into gene translation by separating the actively translating mRNA pool from overall mRNA abundance [3–8], a technique termed TRAP (Translating Ribosome Affinity Purification). Although this approach provides important insights into translational regulation, it lacks the resolution to differentiate between mRNA populations associated with few or high numbers of ribosomes, a distinction that can have major consequences for accurately defining translational rates [9]. This limitation was recently overcome through the development of a technique called “ribosome profiling”, which quantifies only mRNA fragments that are protected by ribosomes (“ribosome footprints”). This enables the quantitative analysis of the number of ribosomes associated with each mRNA transcript, and is even capable of defining regions within RNA transcripts of ribosome association [10, 11]. This technology has been used to reveal genome-wide adaptations to translation that would not have been apparent from transcriptional or translational profiling (TRAP) approaches alone [10, 12–14]. However, despite the potential of ribosome profiling, this approach has not been developed for tissue-specific applications in *Drosophila*.

**Fig 1:**
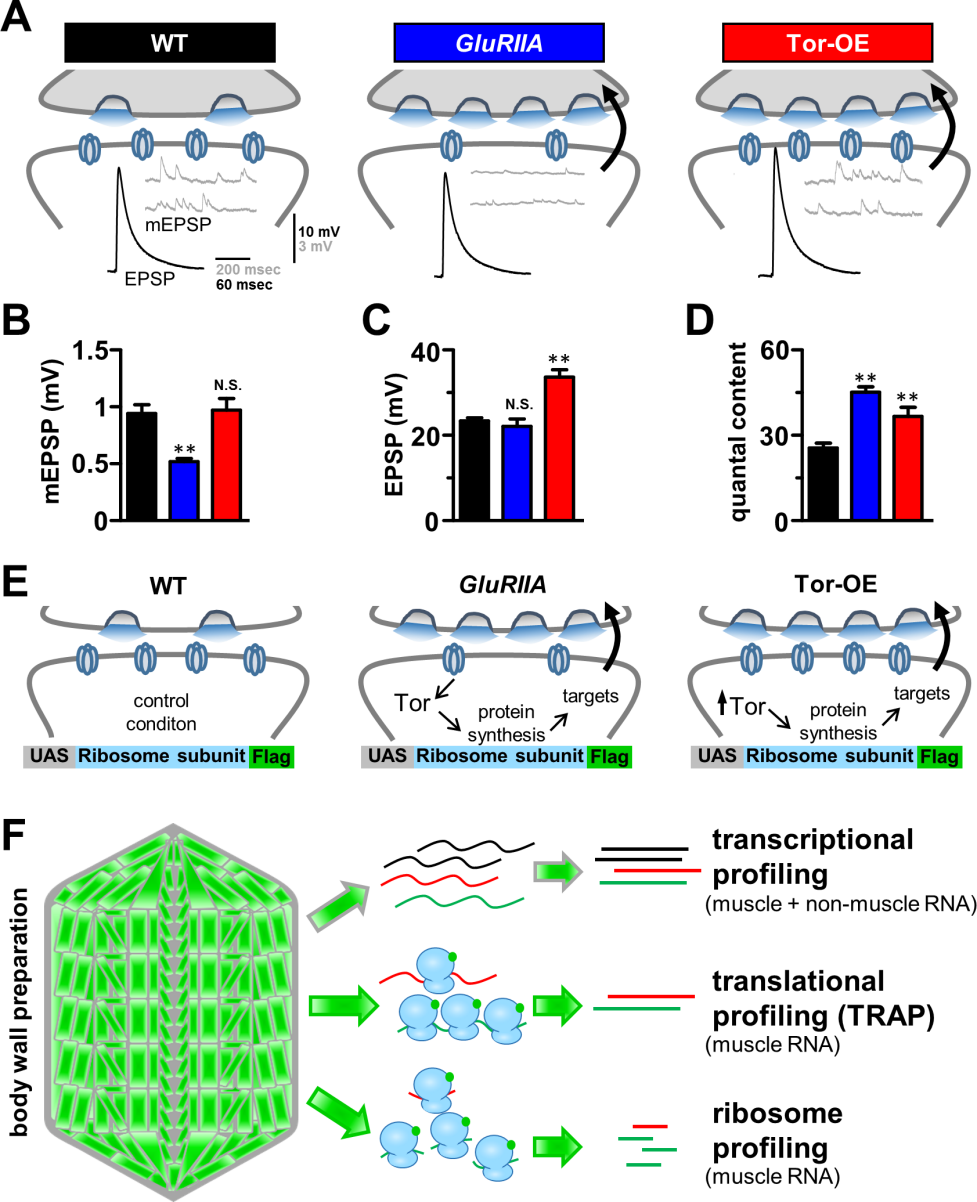
Schematic detailing transcriptional and translational profiling of retrograde homeostatic signaling at the *Drosophila* NMJ. **(A)** Schematic illustrating synaptic transmission at the *Drosophila* NMJ. Representative EPSP and mEPSP electrophysiological traces in wild type (*w*^*1118*^; *BG57-Gal4*/*UAS-RpL3-3xflag*, n = 6), *GluRIIA* mutants (*w*; *GluRIIA*^*SP16*^; *BG57-Gal4*/*UAS-RpL3-3xflag*, n = 6), and overexpression of *Tor* in the postsynaptic muscle (Tor-OE: *w*;*UAS-Tor-myc*/*+*;*BG57-Gal4*/*UAS-RpL3-3xflag*; n = 6). Note that while mEPSP amplitudes are reduced in *GluRIIA* mutants, EPSP amplitudes remain the same as wild type because of a homeostatic increase in presynaptic release (quantal content). Tor-OE does not change mEPSP amplitude, but retrograde homeostatic signaling is induced, leading to increased EPSP amplitude and quantal content. Quantification of mEPSP amplitude **(B)**, EPSP amplitude **(C)**, and quantal content **(D)** for the indicated genotypes. **(E)** Schematic illustrating the putative role of protein synthesis in retrograde homeostatic signaling and the design of ribosome tagging to isolate postsynaptic RNA. **(F)** Schematic representing the workflow for transcriptional profiling, translational profiling using TRAP (translating ribosome affinity purification), and ribosome profiling. Student’s t test was used to compare *GluRIIA* and Tor-OE to wild type; ** = p<0.01.

The *Drosophila* neuromuscular junction (NMJ) is an attractive system to test the power of ribosome profiling. At this model synapse, sophisticated genetic approaches have revealed fundamental genes and mechanisms involved in synaptic growth, structure, function, and plasticity [15, 16]. In particular, translational mechanisms contribute to synaptic growth, function, and plasticity at this synapse [17–19]. Indeed, a key role for translation has recently been implicated in mediating a form of synaptic plasticity intensively studied at the *Drosophila* NMJ referred to as Presynaptic Homeostatic Plasticity (PHP). At this glutamatergic synapse, genetic loss of the postsynaptic receptor subunit *GluRIIA* leads to a reduction in the amplitude of miniature excitatory postsynaptic potentials (mEPSPs; Fig 1A; [20]). However, the amplitude of evoked excitatory postsynaptic potentials (EPSPs) are maintained at wild-type levels due to an enhancement in the number of synaptic vesicles released (quantal content). Thus, a retrograde signaling system is induced by loss of *GluRIIA* that ultimately potentiates presynaptic release, restoring baseline levels of synaptic transmission [21, 22]. Recent forward genetic screening and candidate approaches have revealed the identity of several genes and effector mechanisms in the presynaptic neuron required for the homeostatic potentiation of presynaptic release [22–24]. However, very little is known about the postsynaptic signaling system that transduces a reduction in glutamate receptor function into a retrograde signal that instructs an adaptive increase in presynaptic release. Thus, ribosome profiling of the postsynaptic muscle may reveal the nature of the retrograde signaling system mediating PHP.

There is emerging evidence to strongly suggest that translational mechanisms in the postsynaptic muscle play a key role in retrograde PHP signaling. In particular, pharmacologic or genetic inhibition of postsynaptic protein synthesis through the Target of Rapamycin (Tor) pathway and associated translational modulators disrupts the expression of PHP in *GluRIIA* mutants [25–27]. Interestingly, a constitutive increase in muscle protein synthesis through postsynaptic overexpression of Tor was also shown to be sufficient to trigger the retrograde enhancement of presynaptic release without any perturbation to glutamate receptors [25, 27]. Further, ongoing and sustained postsynaptic protein synthesis is necessary to maintain PHP expression, as acute inhibition of protein synthesis in late larval stages is sufficient to block PHP expression in *GluRIIA* mutants [25, 26]. While these results establish some of the first insights into the postsynaptic signal transduction system controlling retrograde PHP signaling, the putative translational targets involved, and to what extent transcriptional and/or post-translational mechanisms contribute to PHP signaling, remain unknown. Thus, ribosome profiling has the potential to illuminate the translational targets necessary for postsynaptic PHP signal transduction.

We have developed and optimized a streamlined system that enables ribosome profiling from specific tissues in *Drosophila*. We first validate the success of this approach in defining translational regulation in the larval muscle, and reveal dynamics in translation that are distinct from overall transcriptional expression. Next, we highlight the superior sensitivity of ribosome profiling in reporting translational regulation over the conventional TRAP method. Finally, we utilize this ribosome profiling approach to assess translational changes during two cellular processes. First, we evaluate the contributions of transcriptional, translational, and post-translational mechanisms in the postsynaptic muscle that drive the retrograde signaling system underlying presynaptic homeostatic potentiation. Second, we distinguish adaptive changes in transcription and translation that are triggered following a chronic elevation in muscle protein synthesis. This effort has highlighted the unanticipated importance of post-translational mechanisms in ultimately driving retrograde PHP signaling and illuminated the dynamic interplay between modulations in gene transcription and translation as cells acclimate to elevated metabolic activity while maintaining cellular homeostasis.

## Results

### A strategy to profile adaptations in postsynaptic transcription and translation that may drive retrograde PHP signaling

To assess the postsynaptic retrograde signaling systems that drive presynaptic homeostatic potentiation (PHP) at the *Drosophila* NMJ, we focused on three genetic conditions (Fig 1A). First is the wild-type control genotype (*w*^*1118*^;*BG57-Gal4*/*UAS-RpL3-3xflag*), which serves as the control condition in which PHP is not induced or expressed. Second, null mutations in the postsynaptic glutamate receptor subunit *GluRIIA* (*GluRIIA*^*SP16*^;*BG57-Gal4*/*UAS-RpL3-3xflag*) lead to a chronic reduction in mEPSP amplitude [20]. However, EPSP amplitudes are maintained at wild-type levels due to a homeostatic increase in presynaptic release (quantal content) following retrograde signaling from the muscle (Fig 1A–1D). This serves as one condition in which we hypothesized that gene transcription, translation, and/or post-translational changes may have occurred in response to loss of *GluRIIA*, triggering the induction of retrograde signaling that drives PHP. Indeed, in *GluRIIA* mutants, genetic disruption of the translational regulator *Target of rapamycin* (*Tor*) blocks PHP expression, resulting in no change in quantal content and a concomitant reduction in EPSP amplitude [25]. Finally, postsynaptic overexpression of *Tor* in an otherwise wild-type muscle (Tor-OE: *UAS-Tor-myc*/*+*;*BG57-Gal4*/*UAS-RpL3-3xflag*) is sufficient to trigger PHP signaling, leading to increased presynaptic release and EPSP amplitude with no change in mEPSP or glutamate receptors (Fig 1A–1D; [25]). Tor-OE therefore served as the final genotype in which PHP signaling was induced through *Tor* overexpression without any perturbation of postsynaptic glutamate receptors. We hypothesized that changes in translation, and perhaps even transcription, in *GluRIIA* mutants, which may also be apparent in Tor-OE, would illuminate the nature of the postsynaptic transduction system underlying homeostatic retrograde signaling at the *Drosophila* NMJ.

To define genome-wide changes in mRNA transcription and translation in the postsynaptic muscle that may be necessary for PHP signaling, we sought to purify RNA from third instar larvae muscle in wild type, *GluRIIA* mutants, and Tor-OE (Fig 1E). We then sought to define mRNA expression through three methods: Transcriptional profiling, translational profiling using translating ribosome affinity purification (TRAP), and ribosome profiling (Fig 1F). First, we approximated the muscle transcriptome using transcriptional profiling of total mRNA expression by isolating RNA from the dissected third instar body wall preparation. This is primarily composed of muscle tissue, but also contains non-muscle cells including epithelia (Fig 1F). Following extraction of RNA from this preparation, we generated RNA-seq libraries using standard methods (see Materials and Methods). Next, to define translational changes specifically in muscle cells, we engineered an affinity tag on a ribosome subunit under control of the upstream activating sequence (*UAS*), which enables tissue-specific expression (Fig 1E). This biochemically tagged ribosome could therefore be expressed in muscle to purify ribosomes, then processed to sequence only mRNA sequences associated with or protected by ribosomes (Fig 1F). Affinity tagging of ribosomes enabled us to perform translational profiling (TRAP: Translating Ribosome Affinity Purification), an established technique capable of detecting ribosome-associated mRNA [3, 5, 7–9]. Finally, we reasoned that this approach could be optimized to enable ribosome profiling, which has been used successfully to determine changes in translation efficiency, with superior sensitivity over TRAP, in a variety of systems [10, 12, 13, 28]. However, ribosome profiling has not been developed for use in specific *Drosophila* tissues. Our next objective was to optimize a tissue-specific ribosome profiling approach.

### Optimization of a tissue-specific ribosome profiling approach in *Drosophila*

Ribosome profiling is a powerful approach for measuring genome-wide changes in mRNA translation. However, high quantities of starting material is necessary to obtain sufficient amounts of ribosome protected mRNA fragments for the subsequent processing steps involved [29]. Since this approach has not been developed for *Drosophila* tissues, we first engineered and optimized the processing steps necessary to enable highly efficient affinity purification of ribosomes and ribosome protected mRNA fragments by incorporating ribosome affinity purification into the ribosome profiling protocol.

**Fig 2:**
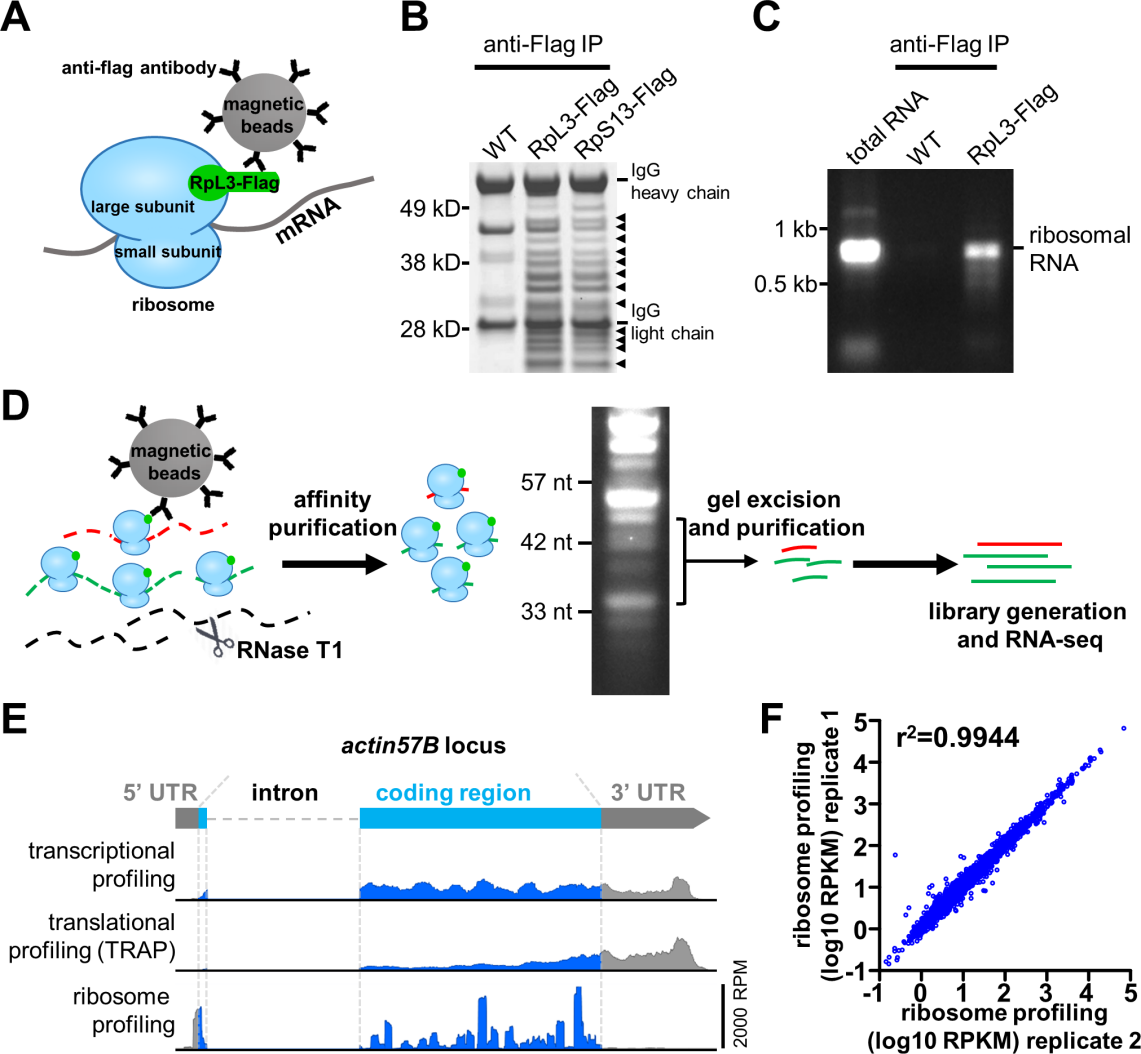
Development and validation of an optimized tissue specific ribosome profiling protocol in *Drosophila*. **(A)** Schematic illustrating the ribosome affinity purification strategy. A tagged ribosome subunit (RpL3-Flag) is expressed and incorporated into ribosomes. Magnetic beads coated with anti-flag antibodies are used to immunoprecipitate ribosomes along with associated mRNAs. **(B)** Anti-flag immunoprecipitation from wild type (control), postsynaptic expression of RpL3-Flag (*w*;*BG57-Gal4/UAS-RpL3-3xflag*), and postsynaptic expression of RpS13-Flag (*w*;*BG57-Gal4/UAS-RpS13-3xflag*) in third-instar larval muscle. Samples were run on an SDS-PAGE gel and Commassie stained. The expected distribution of ribosomal proteins are present in RpL3-Flag and RpS13-Flag samples (noted by arrowheads), but not observed in wild-type controls. **(C)** Total RNA was extracted from anti-flag immunoprecipitations from wild type and RpL3-Flag larval muscle tissue and run on an agarose gel. Ribosomal RNA is present in RpL3-Flag RNA samples but absent in wild type samples. Total RNA extracted from wild type whole larvae was loaded to show the position of ribosomal RNA. **(D)** Workflow for the ribosome profiling strategy. **(E)** Representative RNA-seq mapping of the *actin57B* locus from transcriptional, translational (TRAP), and ribosome profiling. Note that ribosome profiling reads predominantly map to 5’UTR and coding regions, and are absent from the 3’UTR. RPM: reads per million total mapped reads. **(F)** Replicate ribosome profiling sequencing demonstrates highly reproducible results.

Although tissue-specific ribosome affinity purification strategies have been developed before in *Drosophila* [4, 5, 7], these strategies have not been optimized to meet the unique demand necessary for ribosome profiling. Previous approaches tagged the same ribosome subunit (RpL10A) with GFP and 3xflag tags [4, 5, 7], however, we found that these strategies lacked the efficiency necessary for ribosome profiling during pilot experiments. We thus set out to develop and optimize a new ribosome affinity purification strategy that enables the efficient purification and processing of ribosome-protected mRNA. First, we generated transgenic animals that express a core ribosome subunit in frame with a biochemical tag (*3xflag*) under *UAS* control to enable expression of this transgene in specific *Drosophila* tissues (Figs 1E and 2A). Therefore, based on high resolution crystal structures of eukaryotic ribosomes [30], we selected alternative ribosomal proteins from the large and small subunits expected to have C terminals exposed on the ribosome surface. We cloned the *Drosophila* homologs of these subunits, *RpL3* and *RpS13*, in frame with a C-terminal 3xflag tag and inserted this sequence into the pACU2 vector for high expression under *UAS* control [31]. We then determined whether intact ribosomes could be isolated in muscle tissue following expression of the tagged ribosome subunit. We drove expression of *UAS-RpL3-Flag* or *UAS-RpS13-Flag* with a muscle-specific *Gal4* driver (*BG57-Gal4*) and performed anti-Flag immunoprecipitations (Fig 2A). An array of specific bands were detected in a Commassie stained gel from the RpL3-Flag and RpS13-Flag immunoprecipitations, but no such bands were observed in lysates from wild type (Fig 2B). Importantly, identical sized bands were observed in immunoprecipitates from both RpL3-Flag and RpS13-Flag animals, matching the expected distribution of ribosomal proteins [32]. The RPL3-Flag immunoprecipitation showed the same distribution as RpS13 but higher band intensity, indicating higher purification efficiency, so we used *RpL3-Flag* transgenic animals for the remaining experiments. In addition to ribosomal proteins, the other major constituent of intact ribosomes is ribosomal RNA. Significant amounts of ribosomal RNA were detected in an agarose gel from RpL3-Flag immunoprecipitates (Fig 2C), providing additional independent evidence that this affinity purification strategy was efficient at purifying intact ribosomes.

Next, we tested the ability of RpL3-Flag to functionally integrate into intact ribosomes. We generated an *RpL3-Flag* transgene under control of the endogenous promotor (*genomic-RpL3-Flag*; S1A Fig). This transgene was able to rescue the lethality of homozygous *RpL3* mutations (S1A Fig), demonstrating that this tagged ribosomal subunit can integrate and function in intact endogenous ribosomes, effectively replacing the endogenous untagged RpL3 protein. Further, anti-Flag immunostaining of *UAS-RpL3-Flag* expressed in larval muscle showed a pattern consistent with expected ribosome distribution and localization (S1B Fig). Next, we verified that muscle overexpression of RpL3-Flag did not lead to perturbations in viability, synaptic growth, structure, or function (S1C–S1L Fig), nor did muscle overexpression of RpL3-Flag disrupt the expression of PHP in *GluRIIA* mutants or Tor-OE (Fig 1A–1D). Finally, we confirmed that ubiquitous or muscle overexpression of RpL3-Flag did not induce phenotypes characteristic of flies with perturbed ribosome function such as the “minute” phenotype of inhibited growth ([33]; S1A Fig). Thus, biochemical tagging of RpL3 does not disrupt its localization or ability to functionally integrate into endogenous ribosomes.

Finally, we developed and optimized a method to process the isolated ribosomes and generate only ribosome protected mRNA fragments for RNA-seq analysis. First, we digested the tissue lysate with RNaseT1, an enzyme that cuts single stranded RNA at G residues, together with anti-Flag affinity purification (Fig 2D). Following digestion and purification, we ran RNA on a high percentage PAGE gel, excising the mRNA fragments protected from digestion by ribosome binding (30–45 nucleotides in length; Fig 2D). Sequencing of this pool of RNA demonstrated that the vast majority of reads mapped to the 5’UTR and coding regions of mRNA transcripts, with very few reads mapping to the 3’UTR of mRNA transcripts (Fig 2E), where ribosomes are not expected to be associated. This coverage map also revealed heterogeneous distributions on mRNA transcripts with irregular and prominent peaks, as expected, which are indicative of ribosome pause sites on mRNA (Fig 2E; [34]). In contrast, RNA-seq reads for transcriptional and translational profiling using TRAP mapped to the entire mRNA transcript with relatively even coverage (Fig 2E). Extensive metagene analysis confirmed similar distributions around start and stop codons for genome-wide averaged RNA-seq reads (S2 Fig). Importantly, replicate experiments demonstrated that this protocol generated highly reproducible measures of relative protein synthesis rates, defined by mRNA ribosome density, or the number of ribosome profiling Reads Per Kilobase of exon per Million mapped reads (RPKM, also referred to as ribosome profiling expression value; Fig 2F). Thus, expression of *RpL3-Flag* enables the purification of ribosomes from specific tissues in *Drosophila*, and further processing reproducibly generates and quantifies ribosome protected mRNA fragments, which have been demonstrated to correlate with protein synthesis rates [11].

### Ribosome profiling is more sensitive in detecting translational regulation compared to translational profiling (TRAP)

Translation can differ in significant ways from overall transcriptional expression through modulations in the degree of ribosome association with each mRNA transcript, in turn suppressing or enhancing protein synthesis rates [35]. Translation efficiency is a measure of these differences, defined as the ratio of translational to transcriptional expression [10]. Hence, translation efficiency (TE) reflects the enhancement or suppression of translation relative to transcriptional expression due to various translational control mechanisms [36]. Although both translational (TRAP) and ribosome profiling approaches can report TE, ribosome profiling should, in principle, exhibit superior sensitivity in revealing translational dynamics. We therefore compared translational and ribosome profiling directly to test this prediction.

**Fig 3:**
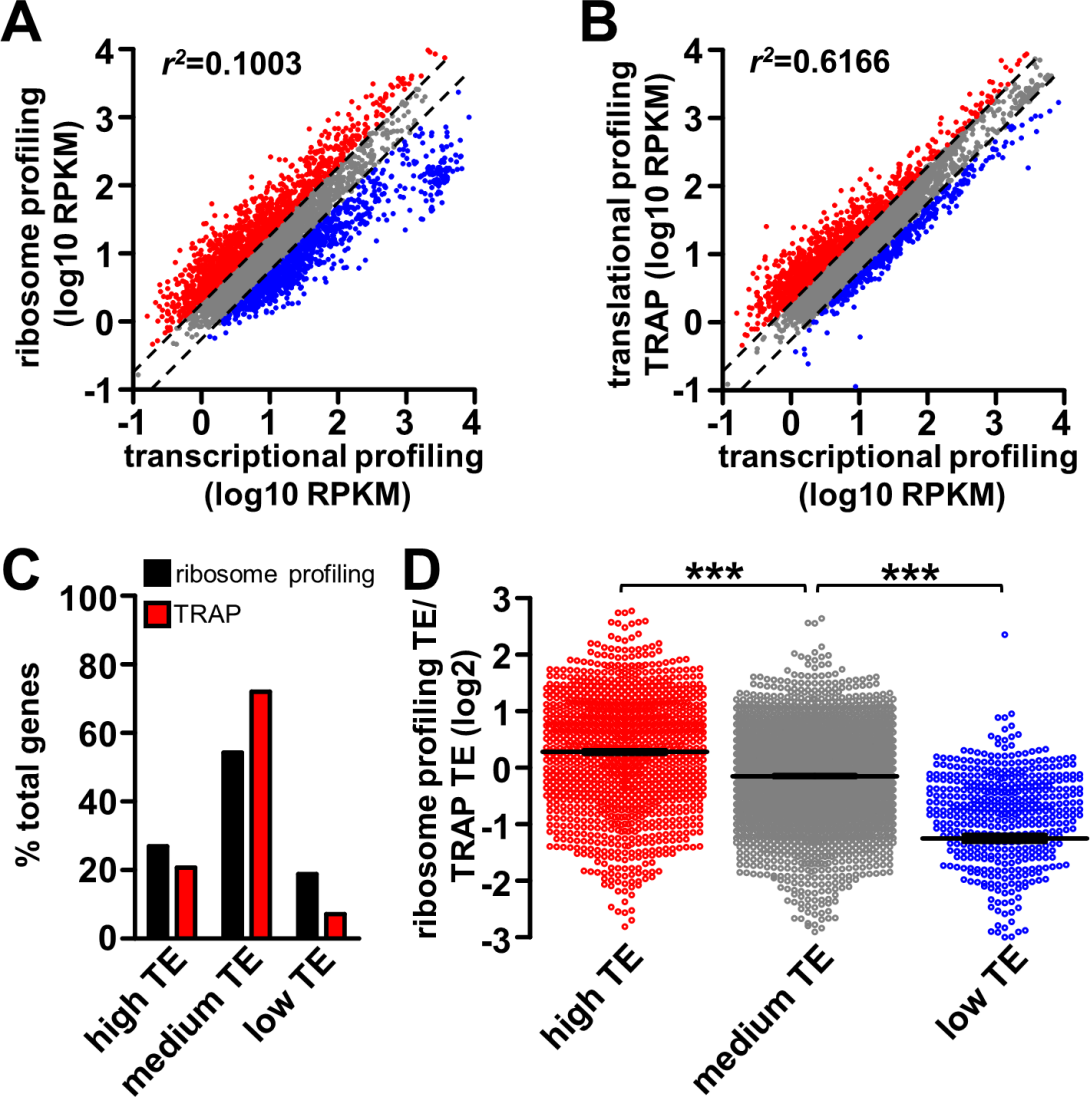
Comparison of translational and ribosome profiling from *Drosophila* larval muscle. **(A)** Plot of ribosome profiling RPKM as a function of transcriptional profiling RPKM for all muscle genes in wild type. Genes with high translation efficiency (TE; TE>2) or low TE (TE<0.5) are labeled in red and blue respectively. Genes with medium TE (TE between 0.5 and 2, indicated by the two dash lines) are labeled in grey. **(B)** Plot of translational profiling (TRAP) RPKM as a function of transcriptional profiling RPKM for all muscle genes in wild type. The same color coding scheme is used as in (A). **(C)** Graph showing percentage of total muscle genes that are in the high TE, medium TE or low TE group based on ribosome profiling or TRAP. Note that a lower percentage of genes are revealed to have high or low TE with TRAP compared to ribosome profiling. **(D)** Plot of translation efficiency defined by ribosome profiling as a ratio of TRAP of all genes in the three categories: high TE (ribosome profiling and TRAP TE average>2), medium TE (TE average between 0.5 and 2), and low TE (TE average<0.5). Note that ribosome profiling reveals higher TE for high TE genes, and lower TE for low TE genes compared to TRAP. *** = p<0.001; one-way ANOVA with post hoc Bonferroni’s test.

We compared TRAP and ribosome profiling to transcriptional profiling in wild-type muscle. In particular, we tested whether differences were apparent in the number of genes revealed to be translationally suppressed or enhanced through ribosome profiling compared to TRAP. We first analyzed the extent to which ribosome profiling and TRAP measurements correlate with transcriptional profiling by plotting the ribosome profiling and TRAP expression values as a function of transcriptional profiling (Fig 3A and 3B; see Materials and Methods). A low correlation would indicate more translational regulation is detected, while a high correlation is indicative of less translational regulation. This analysis revealed a low correlation between ribosome profiling and transcriptional profiling (correlation of determination *r*^*2*^ = 0.100; Fig 3A), while a relatively high correlation was observed between TRAP and transcriptional profiling (*r*^*2*^ = 0.617; Fig 3B). Further, we subdivided all measured genes into three categories: high TE, medium TE, and low TE. These groups were based on translation efficiency as measured by ribosome profiling or TRAP, with high TE genes having a TE value >2, low TE genes having a TE value <0.5, and medium TE genes having a TE between 0.5 and 2. This division revealed a higher number of genes in the high and low TE groups detected by ribosome profiling compared to TRAP (Fig 3C). Together, these results are consistent with ribosome profiling detecting more genes under translational regulation compared to TRAP.

We next investigated the genes under significant translational regulation (genes with high TE or low TE), detected through either ribosome profiling or TRAP, to determine whether differences exist in the amplitude of translational regulation detected. Specifically, genes were divided into the three categories mentioned above based on the average translation efficiency measured by ribosome profiling and TRAP. We then determined the ration of the ribosome profiling TE to TRAP within the three categories. A ratio above 0 (log2 transformed) in the high TE group indicates a more sensitive reporting of translation for ribosomal profiling, while a ratio below 0 in the low TE group would also indicate superior sensitivity for the ribosomal profiling approach. This investigation revealed an average ratio of 0.28 within the high TE group, -0.15 within the medium TE group, and -1.25 within the low TE group (Fig 3D). This analysis demonstrates that ribosome profiling is at least 22% more sensitive in detecting high TE, and 138% more sensitive in detecting low TE in comparison to TRAP. Thus, this characterization demonstrates that ribosome profiling provides a more sensitive and quantitative measurement of translational regulation in comparison to TRAP, validating this approach.

### Transcriptional and ribosome profiling reveals dynamic translational regulation in *Drosophila* muscle

**Fig 4:**
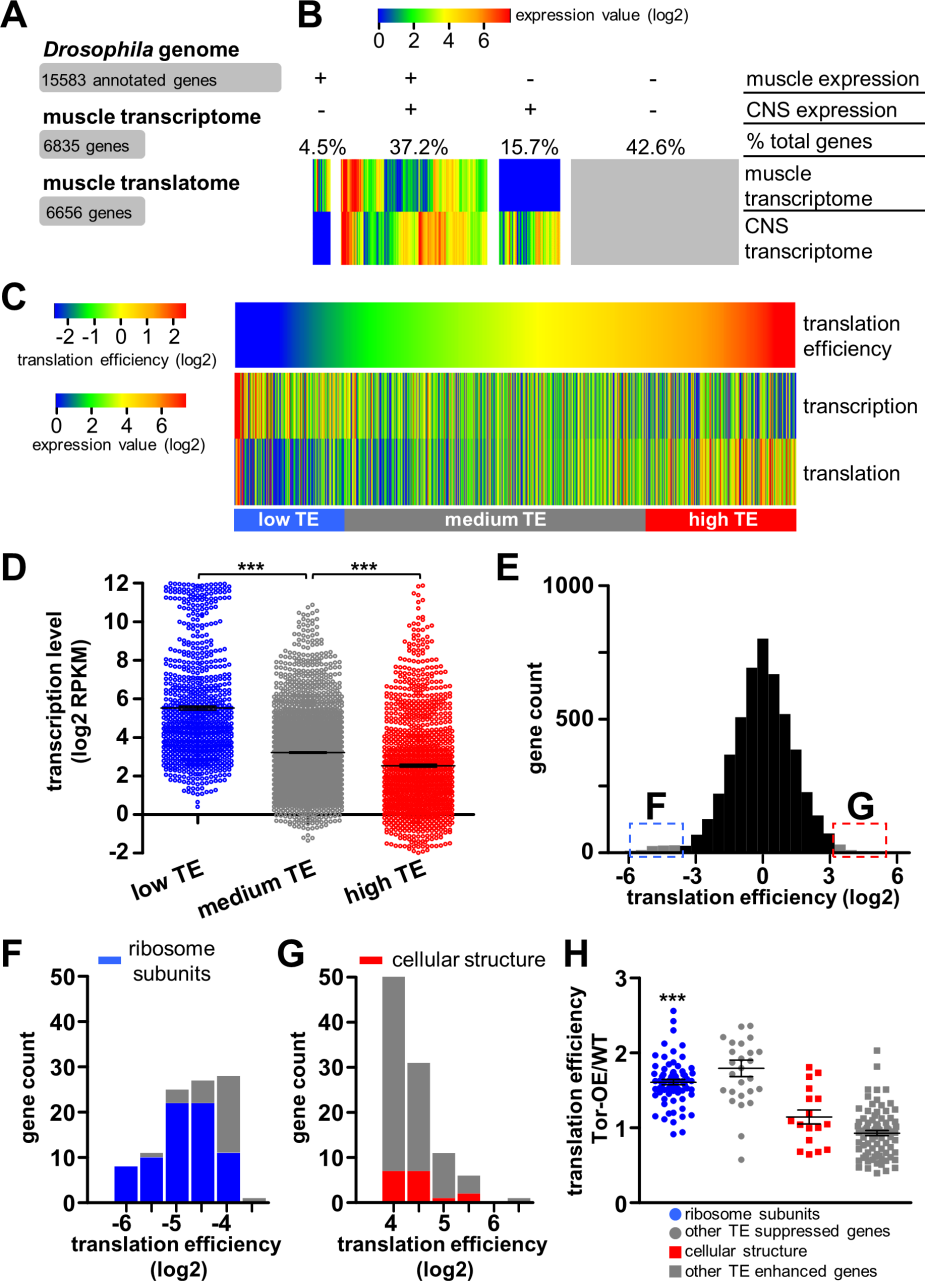
Analysis of the transcriptome and translatome reveals dynamic translational regulation in *Drosophila* muscle. **(A)** Definition of number of genes encoded in the *Drosophila* genome and those expressed in the muscle transcriptome and translatome. **(B)** Heat map showing transcriptional levels of all annotated genes in the *Drosophila* larval muscle compared to those expressed in the central nervous system (CNS; [38]). These genes are grouped into four sections according to their expression status in muscle and CNS; the percentage of total genes is indicated above each section. **(C)** Heat map defining translation efficiency (TE) and transcription and translation expression levels (RPKM) of genes expressed in muscle. Genes are ordered according to translation efficiency, with a trend observed for genes with high translation efficiency having low transcriptional expression levels and vice versa. **(D)** Transcriptional expression levels of genes with low TE (TE<0.5, blue), medium TE (TE between 0.5 and 2, grey), and high TE (TE>2, red). The transcriptional expression levels of genes in the low TE group is significantly higher than that of the medium TE group, while transcriptional expression of the high TE group is significantly lower than that of the medium TE group (*** = p<0.001; one-way ANOVA with post hoc Bonferroni’s test). **(E)** Histogram of translation efficiency across all genes expressed in the muscle. The 100 genes with the lowest translation efficiency (blue) and highest translation efficiency (red) are indicated. **(F)** Histogram of translation efficiency for the 100 genes with the lowest translation efficiency. An enrichment in ribosomal proteins, indicated in blue, is observed. **(G)** Histogram of translation efficiency for the 100 genes with the highest translation efficiency. Genes in the most abundant functional class, encoding proteins involved in cellular structure, are indicated in red. **(H)** Graph showing the TE of the 100 genes with the highest or lowest TE in Tor-OE as a ratio of wild type. Note that the translation efficiency of ribosomal proteins in Tor-OE are significantly increased compared to wild type. *** = p<0.001; paired Student’s t-test. Additional details can be found in S3 and S4 Tables and S3 Fig.

Both subtle and dramatic differences have been observed in rates of mRNA translation relative to transcription, particularly during cellular responses to stress [12, 37]. Having optimized and validated our approach, we went on to perform transcriptional and ribosome profiling in *GluRIIA* mutants and Tor-OE in addition to wild type (S1 Table). To minimize genetic variation, the three genotypes were bred into an isogenic background, and three replicate experiments were performed for each genotype (see Materials and Methods). We first determined the total number of genes expressed in *Drosophila* muscle, as assessed through both transcription and ribosome profiling. The fly genome is predicted to encode 15,583 genes (NCBI genome release 5_48). We found 6,835 genes to be expressed in wild-type larval muscle through transcriptional profiling, and a similar number (6,656) through ribosome profiling (Fig 4A), with ~90% of transcripts being shared between the two lists (S2 Table), indicating that the vast majority of transcribed genes are also translated. A subset of genes that appeared to be transcribed but not translated likely result from non-muscle RNA transcripts derived from the body preparation (see Materials and Methods). Therefore, these transcripts were not analyzed further. We observed no significant differences in the size of the transcriptome and translatome between wild type, *GluRIIA* mutants, and Tor-OE. We then compared the muscle transcriptome to a published transcriptome from the central nervous system (CNS) of third-instar larvae [38]. This analysis revealed dramatic differences in gene expression between the two tissues (Fig 4B). In particular, we found several genes known to be enriched in muscle, including *myosin heavy chain*, *α actinin*, and *zasp52*, to be significantly transcribed and translated in muscle, as expected. In contrast, neural-specific genes such as the active zone scaffold *bruchpilot*, the post-mitotic neuronal transcription factor *elav*, and the microtubule associated protein *tau*, were highly expressed in the CNS but not detected in muscle (S2 Table). Together, this demonstrates that the muscle transcriptome and translatome can be defined by the transcriptional and ribosome profiling strategy we developed with high fidelity.

Next, we investigated genome wide translation efficiency distribution in larval muscle, and compared this with gene expression as assessed through transcriptional and ribosome profiling. We first calculated translation efficiency for all genes expressed in larval muscle and compared heat maps of TE to heat maps of the transcription and translation level (Fig 4C). This revealed a dynamic range of translation efficiency, and a surprising trend of genes with high TE displaying relatively low transcriptional expression levels, while genes with low TE exhibited high transcriptional expression levels (Fig 4C). We then analyzed the genes categorized as high TE, medium TE and low TE (described above) in more detail, comparing the relative distribution in transcriptional expression. We found this trend to be maintained, in that high TE genes exhibited significantly lower transcriptional expression, while low TE genes were significantly higher in transcriptional expression (Fig 4D). Together, this implies a general inverse correlation between translational and transcriptional expression.

Finally, we examined the genes with the most extreme enhancement or suppression of translation efficiency to gain insight into the functional classes of genes that exhibit strong translational control under basal conditions. Interestingly, amongst the genes with the most suppressed translation (100 genes with the lowest translation efficiency), we found a surprisingly high enrichment of genes encoding ribosome subunits and translation elongation factors (Fig 4E and 4F; S3A Fig and S3 Table). Indeed, 73 of the 100 genes with the lowest translation efficiency were ribosome subunits, with all subunits exhibiting a consistently low TE, averaging 0.091. Importantly, we confirmed that overexpression of *RpL3-Flag* does not change transcription of other ribosomal subunits, as quantitative PCR analysis of RpS6 transcript levels were not significantly different between wild type and RpL3-Flag overexpression animals (1.03±0.05 fold compared to wild type, n = 3, p>0.05, Student’s t test). In contrast, *RpL3*, the subunit we overexpressed (*UAS-RpL3-Flag*), was a clear outlier compared with the other ribosome subunits, showing a translation efficiency of 2.85. This was expected due to the *RpL3-Flag* transcript containing artificial 5’ and 3’ UTRs optimized to promote high levels of protein synthesis [31]. This overall suppression in TE of ribosome subunits may enable a high dynamic regulatory range, enabling a rapid increase in production of ribosomal proteins under conditions of elevated protein synthesis. Consistent with this idea, we observed a coordinated upregulation of translation efficiency for ribosomal subunits when overall muscle translation is elevated in Tor-OE (Fig 4H). This is in agreement with previous findings showing ribosome subunits and translation elongation factors as targets for translational regulation by Tor [39, 40]. In contrast to the enrichment of ribosome subunits observed in the low TE group, diverse genes were found among the most translationally enhanced group, with genes involved in cellular structure being the most abundant (Fig 4E and 4G; S3B Fig and S4 Table). These genes may encode proteins with high cellular demands, being translated at high efficiency. Indeed, counter to what was observed in genes with low TE, genes with high TE showed no significant change in TE following Tor-OE (Fig 4H). Together, this analysis reveals that translation differs in dramatic ways from overall transcriptional expression, reflecting a highly dynamic translational landscape in the muscle.

### Transcriptional and ribosome profiling defines genomic deletion in *GluRIIA* mutants and enhanced translation in Tor-OE

**Fig 5:**
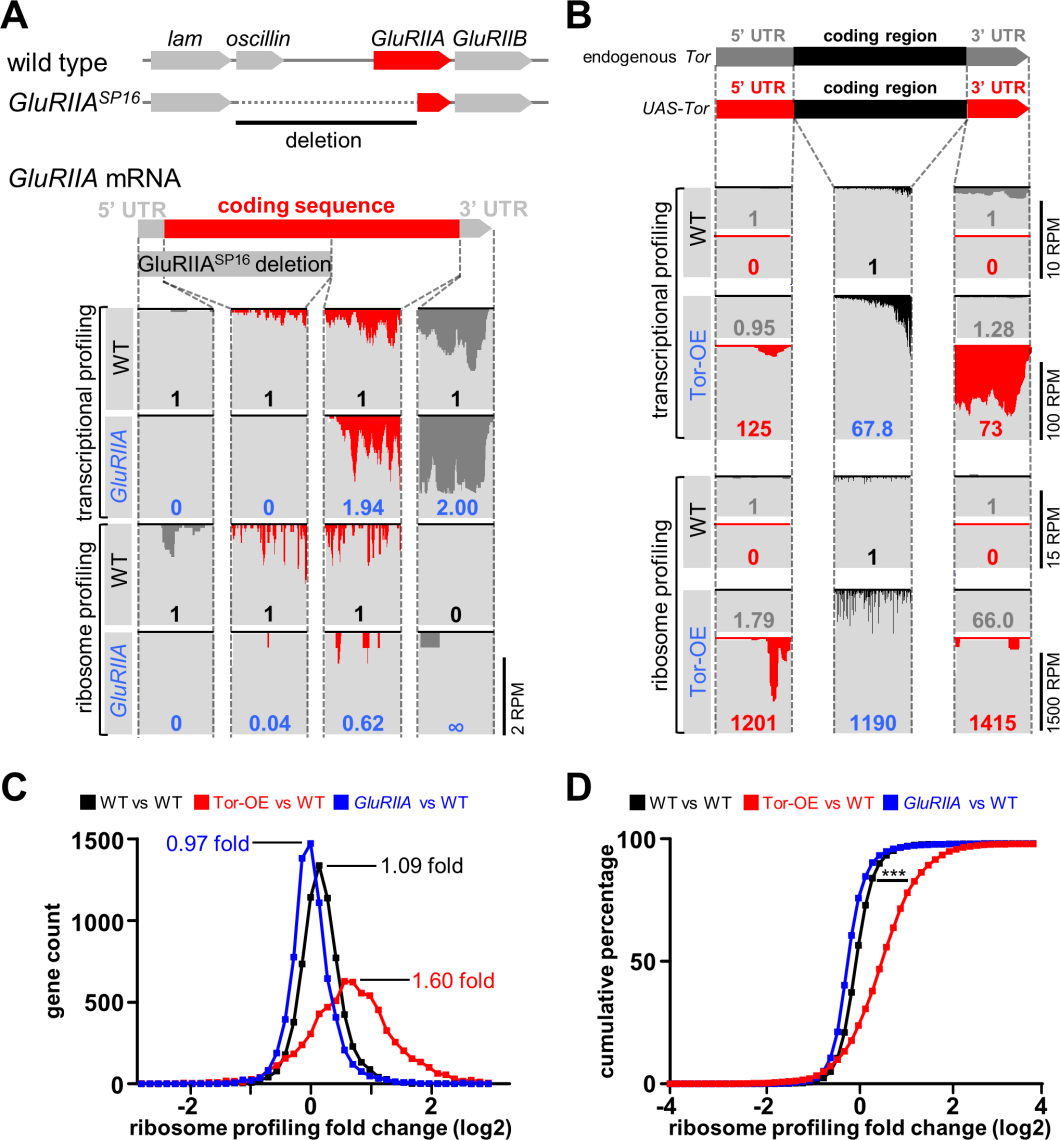
Analysis of transcriptional and translational profiling of *GluRIIA* expression in *GluRIIA* mutants and Tor overexpression in Tor-OE. **(A)** Schematic illustrating the genomic *GluRIIA* locus in wild type and *GluRIIA*^*SP16*^ mutants. Note that the 5’ region of *GluRIIA* is deleted in the *GluRIIA*^*SP16*^ mutant, as well as the adjacent *oscillin* gene. Below: RNA-seq reads mapping to the *GluRIIA* locus from transcriptional and ribosome profiling in wild type and *GluRIIA*^*SP16*^ mutants. The coverage graphs were divided into four sections corresponding to the regions indicated in the *GluRIIA* transcript. The numbers in each graph indicates the expression value of that region normalized to wild type transcriptional or ribosome profiling expression value. Note that no expression was detected by transcriptional or ribosome profiling in the deleted region in *GluRIIA* mutants, as expected. **(B)** Schematic illustrating the endogenous *Tor* mRNA transcript and the mRNA transcript transgenically expressed in Tor-OE (*UAS-Tor*). Both transcripts share the same coding sequence, but differ in their 5’UTR and 3’UTR sequences. Below are reads mapping to the indicated regions, divided into the three indicated sections. Note that both transcriptional and translational expression of *UAS-Tor* mRNA are significantly increased in Tor-OE, while transcription and translation of endogenous Tor mRNA is largely unchanged in Tor-OE. **(C)** Histogram of the distribution of gene translation changes in wild type versus wild type (black), which represents intrinsic variability, that of Tor-OE versus wild type (Red), and that of *GluRIIA* mutants versus wild type (blue). Note the shift in distribution observed in Tor-OE, suggesting a global increase in translation. **(D)** Cumulative percentage plot of distributions shown in (C), showing significant difference between Tor-OE versus wild type distribution compared to wild type versus wild type distribution. (p<0.001, Kolmogorov–Smirnov test).

We confirmed the fidelity of our transcriptional and ribosome profiling approach by examining in molecular genetic detail the two manipulations we utilized to trigger postsynaptic retrograde signaling. The *GluRIIA*^*SP16*^ mutation harbors a 9 kb deletion that removes the first half of the *GluRIIA* locus as well as the adjacent gene, *oscillin* (Fig 5A; [20]). Analysis of both transcriptional and ribosome profiling of *GluRIIA*^*SP16*^ mutants revealed no transcription or translation of the deleted region, as expected (Fig 5A). Transcription and translation of the adjacent gene, *oscillin*, was also negligible (wild type vs. *GluRIIA*: transcription = 15.9 vs. 0.08 RPKM; translation = 9.8 vs. 0.4 RPKM). However, the 3’ portion of the *GluRIIA* coding region was still transcriptionally expressed in *GluRIIA* mutants, while a significant reduction in translation was observed by ribosome profiling (Fig 5A). Together, this confirms that although the residual 3’ region of the *GluRIIA* locus was transcribed, likely through an adjacent promoter, this transcript was not efficiently translated. Indeed, the peak ribosome profiling signals, which represent ribosome pause sites on the mRNA transcript, is known to be conserved for specific open reading frames [34]. However, this pattern was altered in *GluRIIA* mutants compared to wild type (Fig 5A), suggesting the translation of the residual 3’ region of *GluRIIA* in *GluRIIA*^*SP16*^ mutants was not in the same reading frame as the intact transcript. Thus, both transcriptional and ribosome profiling confirms that *GluRIIA* expression is abolished in *GluRIIA*^*SP16*^ mutants.

Next, we examined the expression of endogenous (genomic) and transgenically overexpressed (UAS) *Tor* through transcriptional and ribosome profiling. While both endogenous *Tor* and *UAS-Tor* mRNA share the same coding region, the 5’UTR and 3’UTR regions differ between these transcripts (Fig 5B), enabling us to distinguish expression between these transcripts. We first confirmed a large increase in the expression of the *Tor* coding region in Tor-OE through both transcriptional profiling (68 fold) and ribosome profiling (~1200 fold; Fig 5B, black). In contrast, expression of the endogenous 5’ and 3’ UTRs of *Tor* was similar between *UAS-Tor* and wild type (Fig 5B, grey), while a dramatic increase in the expression of the UTRs of *UAS-Tor* was observed through both transcription (125 fold) and translation (1200 fold; Fig 5B, red). Indeed, the translation efficiency of *Tor* was increased 14 fold in Tor-OE, consistent with the known influences of engineered 5’UTR and 3’UTR sequences in promoting translation in *UAS* constructs [41]. This analysis defines the levels at which Tor transcription and translation are enhanced when *UAS-Tor* is overexpressed in the *Drosophila* larval muscle, and further serve to validate the sensitivity of ribosome profiling.

Finally, we sought to define whether Tor-OE induced a global elevation in translation and to determine whether a similar global shift may have also occurred in *GluRIIA* mutants. Indeed, most if not all mRNAs are capable of being translationally modulated by Tor, with Terminal OligoPyrimidine tract (TOP) mRNAs being the most sensitive to Tor regulation [40, 42]. First, we confirmed a global shift in translation in Tor-OE compared to wild type, as expected given the role of Tor as a general regulator of Cap-dependent translation initiation [43]. We plotted a gene count histogram of Tor-OE versus wild type fold change measured by ribosome profiling, and overlaid the graph over a wild type over wild type ribosome profiling fold change histogram. A shift in global translation was observed in Tor-OE, with an average of 1.6 fold change in translation compared to 1.09 for wild type (Fig 5C). This shift is significant when tested by Kolmogorov–Smirnov test (p<0.001) (Fig 5D). We then performed this same analysis for *GluRIIA* vs WT. However, we observed no significant shift in translation in *GluRIIA* (0.97 fold change compared to 1.09; Fig 5C). Thus, while Tor-OE induces a global increase in translation, loss of the *GluRIIA* receptor subunit in muscle does not measurably change overall translation.

### Transcriptional and ribosome profiling suggest no major differences in transcription or translation contribute to retrograde signaling

Given the substantial evidence that Tor-mediated control of new protein synthesis in the postsynaptic cell is necessary for retrograde PHP signaling [25], we compared transcriptional and translational changes in muscle between wild type, *GluRIIA* mutants, and Tor-OE. We anticipated a relatively small number of transcriptional changes, if any, between these genotypes, while we hypothesized substantial differences in translation would be observed in both *GluRIIA* mutants and Tor-OE. The elevated translation of this exceptional subset of targets would, we anticipated, initiate postsynaptic PHP signaling and lead to an instructive signal that drives the retrograde enhancement in presynaptic release. Alternatively, we also considered the possibility that Tor-mediated protein synthesis may act in a non-specific manner, increasing overall protein synthesis in the postsynaptic cell, while there would be no overlap in translational changes between *GluRIIA* mutants and Tor-OE. In this case, post-translational mechanisms would operate on a global elevation in protein expression in Tor-OE, sculpting the proteome into an instructive retrograde signal. Indeed, the acute pharmacological induction and expression of PHP does not require new protein synthesis [44], providing some support for this model. We therefore compared transcription and translation in wild type, *GluRIIA* mutants, and Tor-OE.

**Fig 6:**
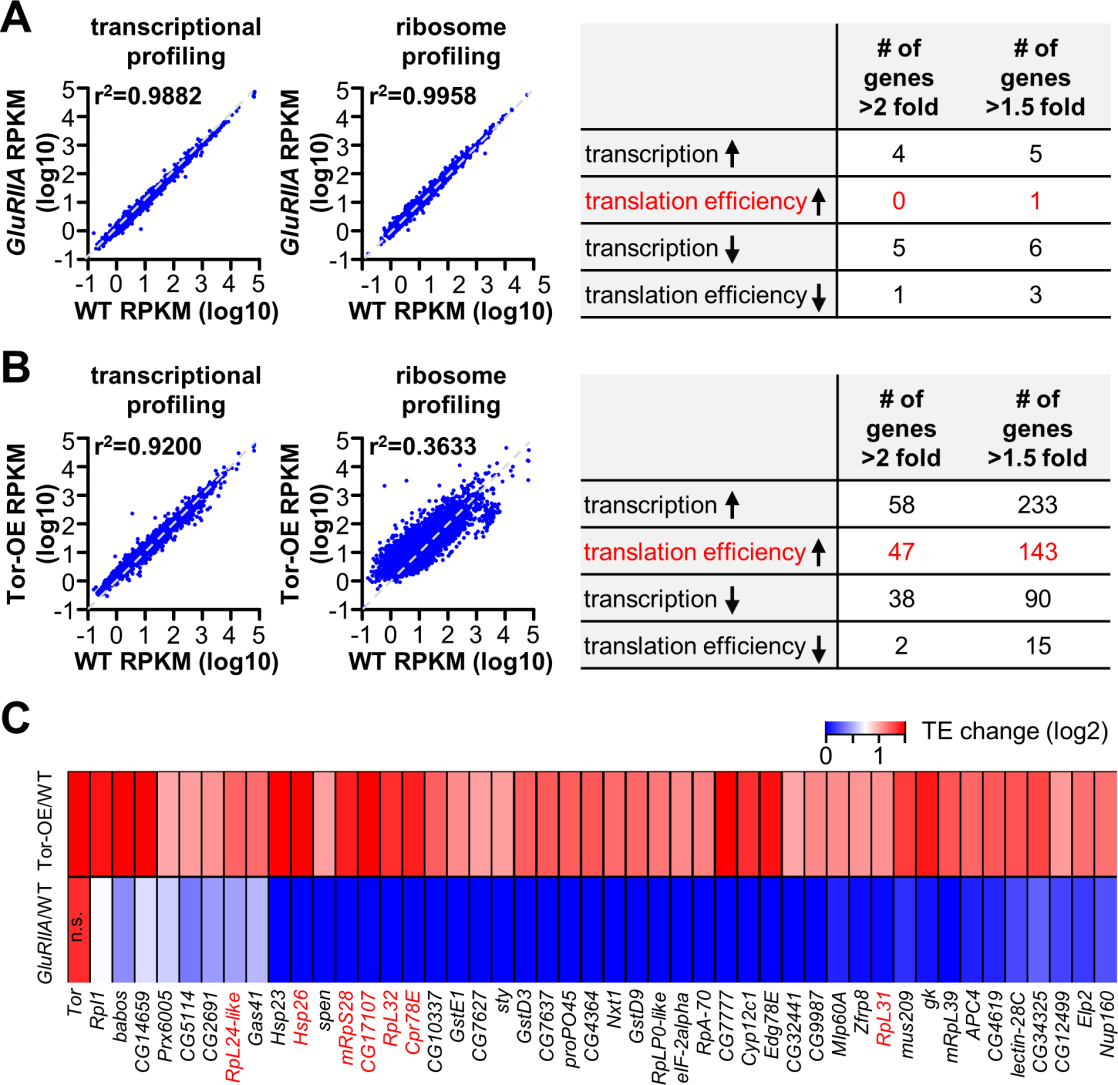
Few changes in postsynaptic transcription or translation are observed in *GluRIIA* mutants. **(A)** Plot of transcriptional and translational expression levels of all genes in *GluRIIA* mutants (*w*^*1118*^;*GluRIIA*^*SP16*^; *BG57-Gal4*/*UAS-RpL3-3xflag*) compared to wild type (*w*^*1118*^; *BG57-Gal4*/*UAS-RpL3-3xflag*), with near perfect correlations observed (indicated by r^2^ values). Right: Table showing the number of genes with significantly up-regulated (up arrow) or down-regulated (down arrow) transcription or translation efficiency (TE) in *GluRIIA* compared to wild type using the indicated cut off values. **(B)** Plot of transcriptional and translational expression levels of all genes in Tor-OE (*w*^*1118*^;*UAS-Tor-myc*/*+*;*BG57-Gal4*/ *UAS-RpL3-3xflag*) compared to wild type. Note that while moderate changes in transcription are observed, large differences in translation are found (indicated by *r*^*2*^ values). Right: Table showing the number of genes with significantly up-regulated (up arrow) or down-regulated (down arrow) transcription or TE in Tor-OE compared to wild type using the indicated cut off values. **(C)** Heat map indicating the 47 genes with significant increase in TE in Tor-OE, with the corresponding genes in *GluRIIA* mutants shown below. Note that no trend is observed in translational expression of these genes in *GluRIIA* mutants. TOP mRNAs are highlighted in red. Additional details can be found in S5 and S6 Tables.

We first compared transcription and translation in *GluRIIA* mutants and Tor-OE relative to wild type by plotting the measured expression values for each condition and determining the coefficient of determination, *r*^*2*^. An *r*^*2*^ value equal to 1 indicates no difference between the two conditions, while a value of 0 implies all genes are differentially expressed. This analysis revealed a high degree of similarity between wild type and *GluRIIA* mutants in both transcription and translation, with *r*^*2*^ values above 0.98 (Fig 6A, left). In contrast, a slightly larger difference exists in transcription between Tor-OE and wild type, with *r*^*2*^ = 0.920 (Fig 6B, left). Although transcription should not be directly affected by Tor-OE, this implies that perhaps an adaptation in transcription was induced in the muscle in response to chronically elevated translation. Finally, translational differences were the largest between Tor-OE and wild type, with *r*^*2*^ values equal to 0.363 (Fig 6B). Indeed, 2,352 genes showed changes greater than 1.5 fold in their measured RPKM compared to wild type in this condition (S6 Table). This global analysis demonstrates there are very few transcriptional and translational changes in *GluRIIA* compared to wild type, while moderate transcriptional and robust translational changes exist in Tor-OE.

Unexpectedly, in depth analysis of the transcriptome and translatome in *GluRIIA* muscle revealed that no genes were significantly altered. In particular, we eliminated genes that were up- or down-regulated due to known or expected influences in the genetic background (*GluRIIA* and *oscillin* expression, and closely linked genes to this locus; see Materials and Methods). Using a standard cut off for expression, we found no gene to have a significant up-regulation in TE more than 2 fold in *GluRIIA* mutants (Fig 6A, right). Even with a lowered threshold for significant expression changes (>1.5 fold change), we observed only 5 genes transcriptionally upregulated and 1 gene translationally upregulated in *GluRIIA* versus WT (Fig 6A, right. S5 Table). Given this small number at such a lowered threshold, we considered the possibility that the genetic background may influence expression of these genes. Consistent with this idea, all 6 upregulated genes are closely linked to the *GluRIIA* locus or were located on the X chromosome, areas we could not fully outcross to the isogenic line (Materials and Methods and S5 Table). Although we cannot rule out transcripts with more subtle differences in translation (below 1.5 fold) or genes with very low and/or highly variable expression that may nonetheless contribute to translational regulation in *GluRIIA* mutants, the sensitivity of ribosome profiling enables us to conclude that no major changes in transcription or translation are present in the postsynaptic muscle of *GluRIIA* mutants.

While no specific translational targets were identified to significantly change in *GluRIIA* mutants, we did identify 47 genes (including Tor itself) that exhibited significant increases in translation efficiency in Tor-OE (>2 fold; Fig 6B, right and S6 Table). Among these 47 genes, 7 encode TOP RNAs [39], including ribosome subunits (Fig 6C). Tor-dependent translational control directly regulates TOP RNAs [39, 40, 42], ribosome profiling was successful in identifying genes in this class. Given the striking finding that very few genes appear to be under transcriptional or translational control in the postsynaptic muscle of *GluRIIA* mutants, we considered the possibility that the 47 genes we identified to be translationally upregulated in Tor-OE may also show a parallel trend in *GluRIIA* mutants but below statistical significance. We therefore generated a heat map of these 47 genes in Tor-OE vs WT and compared this to the same 47 genes in *GluRIIA* vs WT (Fig 6C). This analysis revealed no particular trend or correlation in *GluRIIA* among the 47 genes with increased translation efficiency in Tor-OE (Fig 6C). Together, these results suggest that retrograde signaling in the postsynaptic muscle, induced through loss of *GluRIIA*, does not alter translation of a specific subset of targets, while Tor-OE induces a global, non-specific increase in translation. Thus, post-translational mechanisms are likely to confer the specific signaling processes that ultimately instruct retrograde PHP communication.

### Chronic elevation in muscle protein synthesis leads to adaptive cellular responses

Although Tor -OE is not expected to directly impact transcription, our analysis above indicated that transcriptional changes are induced following the global increase in translation by Tor-OE (Fig 6B). This suggested that adaptations in transcription, and perhaps also translation, may have been triggered in Tor-OE in response to the cellular stress imparted by the chronic, global increase in muscle protein synthesis. Indeed, proteome homeostasis (proteostasis) is under exquisite control [45], and sustained perturbations in Tor activity induces transcriptional programs that adaptively compensate to maintain proteostasis [46, 47]. We therefore reasoned that by examining the changes in transcription and translation induced by Tor-OE, we may gain insight into how a cell adapts to the stress of chronically elevated translation.

**Fig 7:**
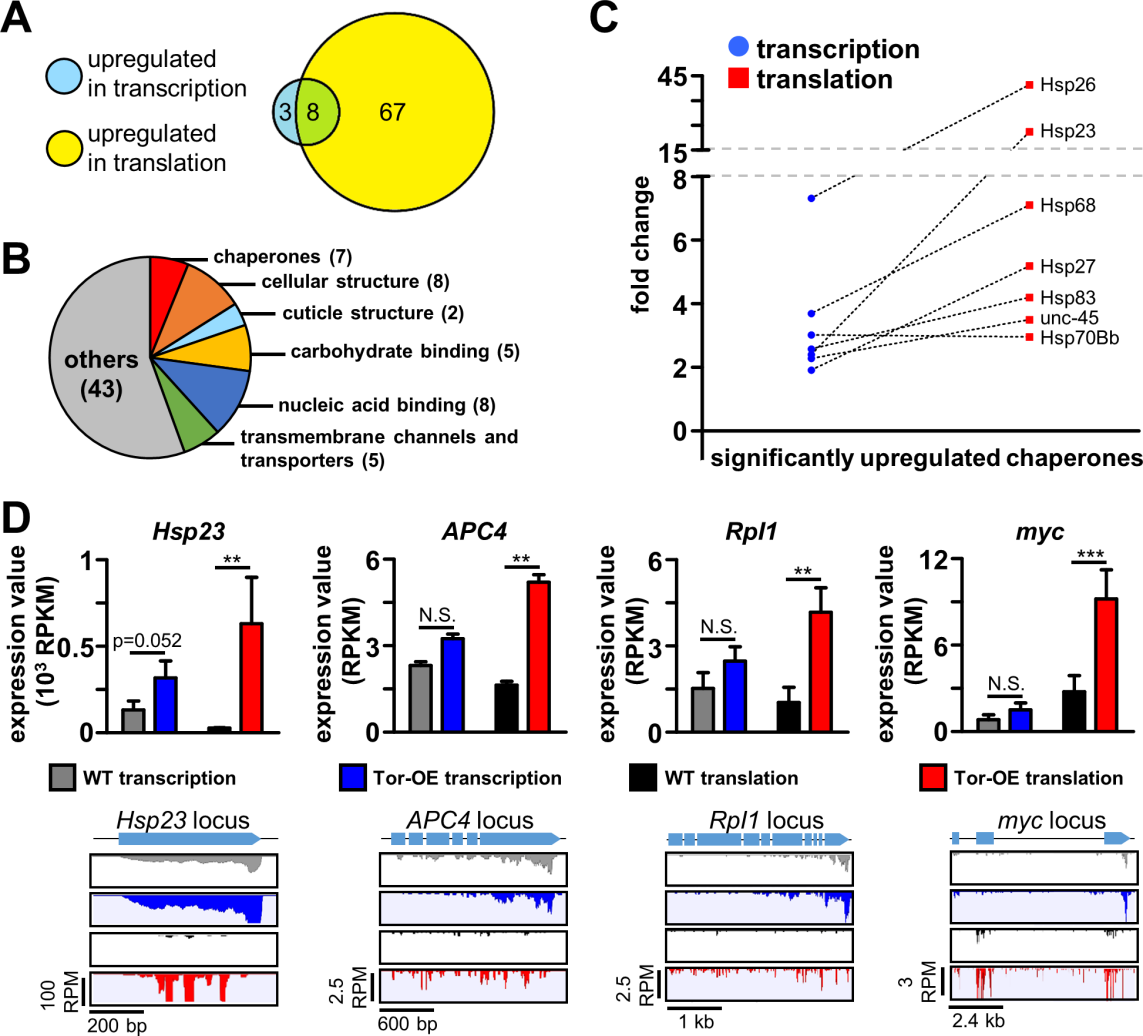
Increased cellular translation triggers adaptive responses in both transcription and translation. **(A)** Diagram showing the number of significantly upregulated genes in transcription and translation in Tor-OE compared to wild type (p<0.05, fold change>3). **(B)** Pie chart illustrating the classes of differentially upregulated genes in Tor-OE compared to wild type. **(C)** Comparative fold changes for chaperones differentially upregulated in transcription and translation in Tor-OE compared to wild type. Note that all but one exhibit higher translational changes compared to transcriptional changes, implying an additional layer of regulation in translation efficiency in addition to the increased transcriptional expression. **(D)** Graphs showing RPKM values measured by transcriptional and ribosome profiling in wild type and Tor-OE for the representative heat shock protein *Hsp23*, the ubiquitin E3 ligase *APC4*, the RNA polymerase *Rpl1*, and the transcription factor *myc*. Read mapping of the indicated genes are illustrated below. ** = p<0.01, *** = p<0.001; Student’s t-test. Additional details can be found in S7 Table.

Transcriptional and ribosome profiling revealed 11 genes with significantly upregulated transcription (fold change>3 and adjusted p-value<0.05; Fig 7A and S7 Table), and 75 genes with significantly upregulated translation (fold change>3 and adjusted p-value<0.05; Fig 7A and S7 Table) in Tor-OE compared to wild type. Interestingly, 8 of these genes exhibited shared increases in both transcription and translation (Fig 7A), with their translational fold change (revealed by ribosome profiling) being larger than would be expected by their transcriptional fold change alone. This suggests a coordinated cellular signaling system that adaptively modulates both transcription and translation in response to the global elevation in translation following overexpression of Tor in the muscle. Further analysis revealed these upregulated genes to belong to diverse functional classes (Fig 7B). Notably, we observed a striking enrichment in genes encoding heat shock proteins and chaperones (GO term fold enrichment of 45.13, p-value = 0.006; GO enrichment test; S4 Fig), factors known to assist with protein folding and to participate in the unfolded protein response, particularly during cellular stress [48, 49]. Indeed, among the 7 heat shock protein genes with significant expression in the muscle (S7 Table), 5 were significantly upregulated in translation and 3 were significantly upregulated in transcription, with the remaining 2 showing a strong trend towards upregulation (Fig 7C and 7D and S7 Table). We performed quantitative PCR as an independent approach to verify the upregulation of heat shock proteins, which confirmed upregulation in the level of total mRNA and ribosome-associated mRNA (S4B and S4C Fig). Given the well documented role for heat shock proteins in regulating protein folding, stability, and degradation in conjunction with the proteasome system [48], this adaptation likely contributes to the stabilization of elevated cellular protein levels resulting from Tor-OE. Thus, the coordinated upregulation of heat shock proteins is one major adaptive response in transcription and translation following Tor-OE.

In addition to heat shock proteins, we also identified genes involved in other cellular functions that are upregulated in Tor-OE and appear to enable adaptive responses to elevated cellular protein synthesis. For example, the E3 ubiquitin ligase subunit *APC4*, involved in proteasome-dependent protein degradation [50], was upregulated in Tor-OE (Fig 7D). Interestingly, proteasome subunits were reported to be upregulated in cells with increased Tor activity [47]. We also identified the RNA polymerase subunit *rpi1* and transcription factor *myc* to be upregulated following Tor-OE (Fig 7D). These genes promote ribosome biogenesis, with RpI1 necessary to synthesize ribosomal RNA and Myc involved in promoting the expression of ribosome assembly factors [51, 52]. Together, RpI1 and Myc likely promote the generation of additional ribosomes to meet the increased demands of protein synthesis induced by Tor-OE, consistent with previous studies showing Tor inhibition leads to decreased RpI1 transcription [53]. Hence, transcriptional and ribosome profiling defined adaptations in gene expression and protein synthesis that maintain proteostasis following chronic elevation in protein synthesis.

## Discussion

We have developed a tissue-specific ribosome profiling strategy in *Drosophila* and used this approach to reveal the transcriptional and translational landscapes in larval muscle. Our analysis revealed significant differences between overall transcriptional and translational expression, and illuminated specific classes of genes with suppressed or elevated levels of translation relative to transcription. We went on to leverage this technology to define the transcriptional, translational, and post-translational influences in the postsynaptic muscle that drive the retrograde control of presynaptic efficacy. Unexpectedly, we found no evidence that specific changes in transcription or translation are necessary for retrograde signaling, indicating that post-translational mechanisms may ultimately transform the loss of postsynaptic receptors and enhanced protein synthesis into instructive retrograde cues. Finally, we identified adaptive cellular responses, in both transcription and translation, to chronically elevated protein synthesis that promote protein stability. Together, this study demonstrates the potential to combine the sophisticated genetic approaches in *Drosophila* with the sensitivity of ribosome profiling to illuminate the complex interplay of transcriptional and translational mechanisms that adaptively modulate cellular proteome stability and trans-synaptic retrograde signaling.

### Ribosome profiling and translational regulation in *Drosophila*

We have developed a highly efficient affinity tagging strategy and optimized RNA processing to enable tissue-specific ribosome profiling in *Drosophila*. Ribosome profiling has major advantages over measuring total mRNA expression and ribosome-associated mRNA (translational profiling using TRAP). Profound differences can exist between transcriptional expression and actual protein synthesis of genes expressed in a tissue. RNA-seq of total mRNA (transcriptional profiling) does not capture translational dynamics [54, 55]. Translational profiling using TRAP does provide insights into translation [9], but is less sensitive in detecting translational dynamics compared to ribosome profiling, which accurately quantifies the number of ribosomes associated with mRNA transcripts (Fig 3; [56]). One major obstacle that limited the development of tissue-specific ribosome profiling is the relatively large amount of starting material necessary to generate the library for next generation sequencing. Because only ~30 nucleotides of mRNA are protected from digestion by an individual ribosome [10], ribosome profiling requires much more input material compared to standard RNA-seq [29]. Thus, the purification efficiency of the ribosome affinity tagging strategy and subsequent processing steps are very important to enabling successful profiling of ribosome protected mRNA fragments in *Drosophila* tissues. We achieved this high purification efficiency by systematically testing and optimizing multiple ribosome subunits (*RpL3*, *RpL36*, *RpS12*, *RpS13*) and affinity tags (6xHis, 1xFlag, 3xFlag), finally settling on the *RpL3-3xflag* combination to enable the highest purification efficiency (Fig 2B and see Materials and Methods). Collectively, this effort differentiates our strategy from previous approaches in *Drosophila* that achieved ribosome profiling but lacked tissue specificity [12] or purified ribosome-associated RNA from specific tissues but lacked the ability to quantify ribosome association with mRNA transcripts [4, 5, 7].

This optimized ribosome profiling approach has illuminated genome-wide translational dynamics in *Drosophila* muscle tissue and demonstrated two opposing protein production strategies utilized in these cells: high transcriptional expression coupled with low translation efficiency, which was apparent for genes encoding ribosomal subunits (Fig 4F), and low transcriptional expression coupled with high translation efficiency, which was observed for genes encoding proteins belonging to diverse functional classes (Fig 4G). These complementary strategies are likely tailored towards different cellular needs, enabling modulatory control of nuclear gene transcription and cytosolic protein synthesis. Thus, transcriptional and ribosome profiling of muscle tissue has revealed that translational control of ribosomal protein synthesis may be a strategy tailored to the unique metabolic needs of this tissue.

### Transcriptional, translational, and post-translational mechanisms during retrograde synaptic signaling

We have used transcriptional and translational profiling to determine the contributions of transcription and translation in the postsynaptic signaling system that drives the retrograde enhancement of presynaptic efficacy. Strong evidence has suggested that protein synthesis is modulated during homeostatic signaling at the *Drosophila* NMJ, with genetic disruption of *Tor*-mediated protein synthesis blocking expression and activation of the *Tor* pathway triggering expression [25–27]. We had expected that translational profiling would discover targets with increased translation efficiency in the muscles of *GluRIIA* mutants and/or following postsynaptic Tor overexpression, genetic conditions in which presynaptic homeostatic plasticity is chronically activated. However, no specific changes in transcription or translation were observed in *GluRIIA* mutants, while a large percentage of muscle genes increased in translation following Tor-OE (Fig 5C and 5D). Furthermore, an apparent global increase in translation also appears sufficient to instruct enhanced presynaptic release, consistent with the nature of the translational regulators implicated in PHP: Tor, S6 Kinase, eIF4E, and LRRK2 [25, 27]. These factors are cap-dependent translational regulators that act on nearly all mRNAs, although there is some degree of differential sensitivity of mRNAs to cap-dependent translational regulation [40, 42]. Although we cannot rule out very subtle changes in translation, nor can we accurately measure levels of transcription or translation in genes with very low or highly variable expression, the sensitivity of the ribosome profiling approach rules out major changes in the translation of specific genes being necessary to promote PHP transduction. Thus, a global enhancement of translation may initiate post-translational mechanisms that are likely to ultimately drive PHP signaling in Tor-OE. Indeed, a recent study demonstrated that *GluRIIA*- and Tor-OE-mediated PHP ultimately converge at a post-translational mechanism to mediate the same retrograde signaling pathway [57]. We consider several possible explanations and implications of these findings.

There are three conditions that trigger homeostatic retrograde signaling in the postsynaptic muscle: Acute pharmacological blockade of GluRIIA-containing postsynaptic receptors [44], genetic mutations in *GluRIIA* [20], and chronic overexpression of Tor [25]. First, all three manipulations lead to a similar enhancement in presynaptic release and converge to drive the same unitary retrograde signaling system [57]. Further, the acute pharmacological induction of PHP does not require new protein synthesis [44, 57]. This implies that while distinct pathways mediate PHP signaling, they all ultimately converge on the same pathway that utilizes post-translational mechanisms. Indeed, there is evidence for post-translational mechanisms in the induction of PHP signaling in *GluRIIA* mutants, as changes in CamKII phosphorylation and activity have been observed [57, 58]. In addition, other post-translational mechanisms, such as protein degradation or ubiquitination, could contribute to homeostatic signaling in the muscle. However, while all three manipulations appear to ultimately utilize the same retrograde signal transduction system, it is quite intriguing that somehow the global increase in translation observed in Tor-OE is sculpted, perhaps by shared post-translational mechanisms, into a specific retrograde signal that instructs enhanced presynaptic release.

Second, it is possible that pharmacological, genetic, or Tor-OE-mediated inductions of PHP signaling are all mechanistically distinct, in which case no common transcriptional, translational, or post-translational mechanisms would be expected. Indeed, forward genetic screening approaches to discover genes necessary for PHP expression have failed to identify any genes needed for PHP induction in the postsynaptic muscle [23, 59], suggesting possible redundancy in these signaling systems. Further, it is possible that very small, local changes in translation are necessary to drive retrograde signaling in *GluRIIA* mutants and Tor-OE, in which case our ribosome profiling approach may have lacked sufficient resolution to detect these changes, as tagged ribosomes were purified from whole muscle lysates. Indeed, a recent report demonstrated synapse-specific PHP expression [58]. Future studies utilizing genetic, electrophysiological, biochemical, and imaging approaches will be necessary to identify the specific post-translational mechanisms that drive PHP signaling, and to what extent shared or distinct mechanisms are common between pharmacologic, genetic, and Tor-OE mediated PHP signaling.

### Proteostasis and adaptive cellular responses to elevated protein synthesis

Cells possess a remarkable ability to homeostatically control protein expression and stability, a process called proteostasis [60]. This requires a robust and highly orchestrated balance between gene transcription, mRNA translation, and protein degradation [45, 61], while disruption of this process contributes to aging and disease [62, 63]. Further, proteostatic mechanisms are not only customized to the unique demands of specific cells and tissues, but are adjusted throughout developmental stages and even tuned over hours according to diurnal metabolic and feeding cycles [64–66]. The homeostatic nature of proteostasis is highlighted by the adaptations triggered in response to perturbations that threaten stable cellular protein levels, such as starvation and inhibitions of protein degradation [67, 68]. We have used transcriptional and ribosome profiling to reveal new homeostatic adaptations triggered by proteostatic mechanisms that stabilize the proteome following chronic elevations in protein synthesis. In particular, genes that promote protein stability (chaperones), protein degradation, and ribosome biogenesis were transcriptionally and/or translationally upregulated following *Tor* overexpression in muscle (Fig 7), modulations in complementary pathways that synergistically prevent inappropriate protein interactions, promote protein removal, and increase the machinery necessary to maintain elevated protein synthesis [47, 53, 69]. Interestingly, many of these pathways are also targeted following other homeostatic perturbations to proteome stability, including heat shock, starvation, and inhibitions in protein degradation [67, 70]. This may suggest that proteostatic signaling involves a core program orchestrating adaptive modulations to transcription and translation in response to a diverse set of challenges to protein stability. Thus, ribosome profiling enabled the definition of transcriptional and translational mechanisms that respond to chronic elevations of protein synthesis, revealing changes in translation that would not be apparent through profiling of total RNA expression alone.

Recent developments in next-generation sequencing have greatly expanded our ability to investigate complex biological phenomena on genome-wide scales. The power and variety of sophisticated genetic approaches are well-known in *Drosophila*. These include tissue-specific expression with a broad array of Gal4 and LexA drivers, transposable element manipulations, CRISPR/Cas-9 gene editing, and extensive collections of genetic mutations and RNAi lines [71–74]. Although some approaches have emerged that permit the analysis of RNA from entire organs as well as ribosome-associated RNA from specific tissues [5–7, 9, 38, 75, 76], the technology described here now adds ribosome profiling to join this powerful toolkit to enable the characterization of translational regulation in specific cells with unprecedented sensitivity.

## Materials and methods

### Fly stocks and molecular biology

*Drosophila* stocks were raised at 25°C on standard molasses food. The *w*^*1118*^ strain is used as the wild type control unless otherwise noted, as this is the genetic background of the transgenic lines and other genotypes used in this study. The following fly stocks were used: *GluRIIA*^*SP16*^ [20], *UAS-Tor-myc* [77], *RpL3*^*G13893*^ (Bloomington Drosophila Stock Center, BDSC, Bloomington, IN, USA), *RpL3*^*KG05440*^ (BDSC). All other *Drosophila* stocks were obtained from the BDSC. To control for the effects of genetic background on next generation sequencing data, we generated an isogenic stock and bred the genetic elements used in this study, (*BG57-Gal4*, *UAS-RpL3-Flag*, *GluRIIA*^*SP16*^, and *UAS-Tor-myc*) into this isogenic line by outcrossing for five generations to minimize differences in the genetic background.

During initial testing phases to determine the optimal ribosome subunit and biochemical tag to use, we generated several constructs and systematically compared purification efficiency. In particular, we inserted 1xFlag-6XHis tags to the C-terminals of the ribosome subunits RpL3, RpL36, RpS12, and RpS13. We engineered expression with each subunit’s genomic promotor into the pattB vector [78]. Transgenic stocks were made and tested for affinity purification of intact ribosomes using cobalt ion-coupled beads (Clontech, 635501). These biochemical tags were found to be inferior when compared to a single 3xFlag tag, which was used for the design of all subsequent constructs. To generate the *UAS-RpL3-3xFlag* and *UAS-RpS13-3xFlag* transgenic lines, we obtained cDNA containing the entire coding sequences of *RpL3* (FBcl0179489) and *RpS13* (FBcl0171161). *RpL3* and *RpS13* coding sequence were PCR amplified and sub-cloned into the pACU2 vector [31] with C-terminal 3xflag tag using a standard T4 DNA ligase based cloning strategy. To generate the genomic RpL3-3xflag construct, a 6.5kb sequence containing the entire *RpL3* genomic locus was PCR amplified from a genomic DNA preparation of *w*^*1118*^ using the following primers 5’-ATCGGTACCACTTACTCCCTTGTTG-3’ and 5’-CAGCTGCAGGGTTTGTGACTGATCTAAAAG-3’. The same linker-3xflag sequence used in *UAS-RpL3-3xflag* was inserted before the stop codon of *RpL3* using extension PCR. This sequence was cloned into the pattB vector [78]. Constructs were sequence verified and sent to BestGene Inc. (Chino Hills, CA) for transgenic integration.

### Affinity purification of ribosomes and library generation

#### Tissue collection, lysis, and library generation for transcriptional profiling

18 female third-instar larvae for each preparation were controlled for developmental stage by selection within 3 hours of emerging from media [79]. Larvae were dissected in HL-3 saline as previously described [80], with all internal organs and the central nervous system removed, leaving only the body wall and attached muscle tissue. Following dissection, the tissue was immediately frozen on dry ice. The frozen tissue was then homogenized in 540 μl lysis solution (10 mM HEPES, PH 7.4, 150 mM KCl, 5 mM MgCl_2_, 100 μg/ml Cycloheximide) supplemented with 0.5% Triton-X100, 1U/μl ANTI-RNase (ThermoFisher scientific, AM2690) and protease inhibitor (EDTA-free, Sigma, COEDTAF-RO). 120 μl of the lysate was used for total RNA extraction by TRIzol LS Reagent (ThermoFisher scientific, 10296010). 2.5 μg of total RNA was used for isolation of mRNA with the Dynabeads mRNA DIRECT Purification Kit (ThermoFisher scientific, 61011). The entire isolated mRNA sample was used for library generation with the NEBNext Ultra Directional RNA Library Prep Kit for Illumina sequencing (NEB, E7420S). 15 PCR cycles were used in the amplification step of all library generation protocols, as no significant PCR duplicates were observed in the sequencing results of the quality control step. We used whole body wall tissue as an approximation for the total muscle RNA preparation used for transcriptional profiling, as muscle is the dominant tissue in this preparation.

#### Purification of ribosome associated mRNA and library generation (TRAP)

180 μl of the lysate described above was incubated with anti-flag antibody coated magnetic beads to purify ribosomes with associated mRNA. 75 μl of Dynabeads protein G (ThermoFisher scientific, 10004D) was used to coat 3 μg anti-Flag antibody (Sigma, F1804). The lysate-beads mixture was incubated at 4°C with rotation for 2 hours, then washed in buffer (10 mM HEPES, PH 7.4, 150 mM KCl, 5 mM MgCl_2_, 100 μg/ml Cycloheximide), supplemented with 0.1% Triton-X100 and 0.1 U/μl SUPERase in RNase Inhibitor (ThermoFisher scientific, AM2696). RNA was extracted from ribosomes bound to the beads by TRIzol Reagent, and the co-precipitant linear acrylamide (ThermoFisher scientific, AM9520) was used to increase the RNA recovery rate. mRNA isolation and library generation were performed as described above.

#### Library generation for ribosome profiling

240 μl of lysate was incubated with anti-Flag antibody coated magnetic beads and 10000 units of RNase T1 (ThermoFisher scientific, EN0541) to perform digestion of exposed mRNA and ribosome purification simultaneously. 100 μl of Dynabeads protein G coated with 4 μg anti-Flag antibody was used. The lysate-beads-RNase T1 mixture was incubated at 4°C for 6 hours and washed; RNA was extracted as described above.

To perform size selection, the extracted RNA sample was separated on a denaturing 15% polyacrylamide urea gel. The gel region corresponding to 30–45 nt, as estimated by oligo markers, was excised. The gel slice was homogenized in 500 μl elution buffer (10 mM Tris-HCl, PH 7.5, 250 mM NaCl, 1 mM EDTA) supplemented with 0.2% SDS and RNA*secure* reagent (ThermoFisher scientific, AM7005). The gel slurry was heated at 60°C for 10 min to allow inactivation of contaminating RNase by RNA*secure* reagent and transferred to 4°C for overnight elution of RNA from the gel. The eluate was collected by centrifuging the gel slurry through a Spin-X column (Sigma, CLS8162), and RNA was precipitated by adding an equal volume of isopropanol and 25 μg linear acrylamide, incubated at room temperature for 30 min, and centrifuged for 15 min at 17000Xg, 4°C. The pellet was air dried and dissolved in 15 μl RNase-free water.

#### Library generation for ribosome profiling was performed using NEBNext Small RNA

Library Prep Set for Illumina (NEB, E7330S) with minor modifications. The entire size selected mRNA fragments sample were first treated by phosphatase, rSAP (NEB, M0371S), to remove the 3’-phosphate. The samples were then incubated and denatured according to manufacturer’s instructions. RNA was precipitated from the reaction as described above, and the 3’ adaptor ligation was performed using NEBNext Small RNA Library Prep Set. The 5’-phosphate was then added to the mRNA fragments by supplying 2.5 μl 10 mM ATP, 1.5 μl 50 mM DTT and 0.5 μl T4 Polynucleotide Kinase (NEB, M0201S) to the 20 μl 3’ adaptor ligation reaction and incubating at 37°C for 30 min. 1 μl SR RT primer of the NEBNext Small RNA Library Prep Set was then added to the T4 polynucleotide kinase reaction and RT primer hybridization was performed. 5’ adaptor ligation, reverse transcription, PCR amplification and size selection of the PCR amplified library were performed using the NEBNext Small RNA Library Prep Set. A detailed protocol is deposited at www.protocols.io (dx.doi.org/10.17504/protocols.io.j89crz6).

### High-throughput sequencing and data analysis

All libraries were sequenced on the Illumina NextSeq platform (single read, 75 cycles), and three replicates were performed for each genotype. All sequencing datasets are deposited in the NCBI GEO datasets, accession number: GSE99920. Sequencing data analysis was performed using CLC genomics Workbench 8.0 software (Qiagen). Raw reads were trimmed based on quality scores, and adaptor sequences were removed from reads. Trimmed high quality reads were then mapped to the *Drosophila* genome (*Drosophila melanogaster*, NCBI genome release 5_48). Only genes with more than 10 reads uniquely mapped to their exons were considered to be reliably detected and further analyzed, as the variability was sharply higher for genes with less than 10 mapped reads compared to genes with mapped reads above 10 (S5 Fig). We excluded genes from further analysis that were only found to be transcriptionally expressed, which were likely to result from non-muscle RNA. Relative mRNA expression levels were quantified by calculating RPKM (Reads Per Kilobase of exon per Million mapped reads) using mapping results from transcriptional profiling. Relative translation levels were quantified by calculating RPKM using mapping results from ribosome profiling. Translation efficiency was calculated by dividing ribosome profiling (or translational profiling TRAP) RPKM by transcriptional profiling RPKM.

To determine differentially transcribed or translated genes, a weighted t-type test [81] was performed based on three replicate expression values for each gene between *GluRIIA* mutants and wild type, and Tor-OE and wild type using the statistical analysis tool of CLC genomics workbench. The analysis was performed on expression values obtained by transcriptional profiling to determine differentially transcribed genes, and on expression values obtained by ribosome profiling to determine differentially translated genes. Genes with a p-value less than 0.05 and fold change higher than 3-fold were considered differentially transcribed or translated unless otherwise stated. We also determined differentially transcribed or translated genes using R package DESeq2 analysis [82], considering genes with adjusted p-values less than 0.05 as differentially expressed. The Baggerly’s t test method and DESeq2 method produced highly similar lists of differentially expressed genes. To determine gene targets undergoing translational regulation in *GluRIIA* mutants and Tor-OE compared to wild type, two criteria were used. First, the gene must have at least a 2-fold significant increase (p<0.05, Student’s t test) in translation efficiency compared to wild type. Second, a significant increase in ribosome profiling expression value (p<0.05, Baggerly’s t test) must also exist for the same gene. These two criteria ensure identification of genes that have true translational up-regulation that are not due to transcriptional changes. Metagene analysis was performed using Plastid analysis software [83] using default settings.

### Immunocytochemistry and confocal imaging

Third-instar larvae were dissected in ice cold 0 Ca^2+^ HL-3 and fixed in Bouin's fixative for 2 min and immunostained and imaged as described [84].

### Quantitative PCR

Quantitative PCR (qPCR) was performed using Luna® Universal One-Step RT-qPCR Kit (NEB, E3005S) according to manufacturer’s instructions. RNA was isolated and prepared from body wall tissue as described above. 5 ng of total RNA was used as template in each reaction. Three biological replicates were performed for each sample and the 2^^-ΔΔCt^ method was used for qPCR data analysis. The primers used for assaying each target are as follows (fwd/rev, 5’-3’):

*Hsp83*: GCAGCGTCTGAAAAGTTTTGTG; AATCTCAGCCTGGAATGCA.
*Hsp68*: ACCATCAAGAACGACAAGGG; ATAGGTCTCCAGTTGATTGCG.
*Hsp26*: CTCACCGTCAGTATTCCCAAG; CCTTCACCTCGCTTTCATTTG.
*αTub84D* (control): CTACAACTCCATCCTAACCACG; CAGGTTAGTGTAAGTGGGTCG.
*RpS6*: ATGAAGCAGGGTGTCTTGAC; AGAGCCAGCACAGACATG.

### Electrophysiology

All recordings were performed in modified HL-3 saline with 0.3 mM Ca^2+^ as described [85].

### Statistical analysis

All data are presented as mean +/-SEM. A Student’s t test was used to compare two groups. A one-way ANOVA followed by a post-hoc Bonferroni’s test was used to compare three or more groups. All data was analyzed using Graphpad Prism or Microsoft Excel software, with varying levels of significance assessed as p<0.05 (*), p<0.01 (**), p<0.001 (***), N.S. = not significant. Statistical analysis on next generation sequencing data was described in the *High-throughput sequencing and data analysis* section.

## Supporting information

S1 Fig *RpL3-Flag* can restore viability to RpL3 mutants and does not perturb synaptic growth or function when overexpressed. **(A)** Schematic illustrating the genomic *RpL3-Flag* rescue construct and table showing the lethal phase of *RpL3* mutants (*w*;*RpL3*^*G13893/KG05440*^) compared to RpL3-Flag rescue (*w*;*genomic-RpL3-3xflag*/+; *RpL3*^*G13893/KG05440*^). **(B)** Representative images of larval muscles of BG57>RpL3-Flag (muscle overexpression of *RpL3-Flag*; *w*;*BG57-Gal4/UAS-RpL3-3xflag*) immunostained with anti-Flag (green) and anti-HRP (neuronal membrane marker; magenta) antibodies. **(C)** Representative images of muscle 6/7 NMJs from wild type (*w*^*1118*^) and BG57>RpL3-Flag immunostained with antibodies against vGlut (synaptic vesicle marker; magenta) and HRP (white). **(D)** Quantification of bouton number in the indicated genotypes. **(E)** Individual boutons of wild type and BG57>RpL3-Flag NMJs immunostained with antibodies against BRP (active zone marker; green), GluRIID (postsynaptic glutamate receptor marker; magenta), and DLG (postsynaptic density marker, white). No significant differences are observed in BRP number per NMJ **(F)**, Dlg intensity **(G)**, or GluRIID intensity **(H)** in wild type (n = 12) and BG57>RpL3-Flag (n = 12). **(I)** Representative traces of EPSP and miniature EPSP recordings from wild type and BG57>RpL3-Flag third-instar larval NMJs. No significant differences are observed in miniature EPSP frequency **(J)**, miniature EPSP amplitude **(K)**, or EPSP amplitude **(L)** in wild type (n = 11) and BG57>RpL3-Flag (n = 15). N.S = p>0.05; Student’s t test.
(TIF)

S2 Fig Comparative metagene analysis of genome-wide averaged reads distribution around the start and stop codons from transcriptional, translational (TRAP) and ribosome profiling. **(A)** Metagene analysis plot of averaged read density around the start codon for transcriptional, translational (TRAP) and ribosome profiling. **(B)** Metagene analysis plot of averaged read density around the stop codon for transcriptional, translational (TRAP) and ribosome profiling. Note the highly reduced density of ribosome profiling reads in the 3’UTR. UTR: untranslated region.
(TIF)

S3 Fig Functional classes for the 100 genes with the lowest and highest translation efficiency (TE). **(A)** Functional classes for the 100 genes with the lowest TE. Note that ribosomal proteins represent the largest class, with 73 of the 100 genes encoding ribosomal proteins. **(B)** Functional classes for the 100 genes with the highest TE. Diverse functional classes are present, with genes encoding proteins involved in cellular structure being the most abundant class.
(TIF)

S4 Fig GO analysis and validation of transcriptional and translational upregulation of heat shock proteins in Tor-OE using quantitative PCR. **(A)** GO term enrichment analysis of genes up-regulated in Tor-OE; a GO enrichment test is used to generate the p-values indicated. **(B)** Quantitative PCR (qPCR) analysis of three heat shock proteins (Hsp83, Hsp68, Hsp26) from wild type (*w*^*1118*^; *BG57-Gal4*/*UAS-RpL3-3xflag*) and Tor-OE (*w*^*1118*^;*UAS-Tor-myc*/*+*;*BG57-Gal4*/ *UAS-RpL3-3xflag*) larvae. Total RNA from preparations of each genotype was used as the template for the qPCR assay to assess transcriptional changes. **(C)** qPCR analysis of the indicated heat shock proteins and genotypes using ribosome associated RNA from the preparations as the qPCR template to assay translational changes. *** = p<0.001; Student’s t-test.
(TIF)

S5 Fig Effect of mapped read number on transcriptional and ribosome profiling measurement variability. **(A)** Number of genes (of 15,583 total genes encoded in the *Drosophila* genome) that fall into each indicated read number range for transcriptional profiling and ribosome profiling. **(B)** Transcriptional profiling variability, defined as the standard error of the mean (SEM) normalized to the mean RPKM value of each gene, as a function of the number of mapped reads. Mapped reads for each gene were grouped and binned at indicated values. **(C)** The same plot as in B for reads obtained through ribosome profiling. Note that variability is sharply increased at read numbers below 10 in both transcriptional and ribosome profiling.
(TIF)

S1 Table Analysis of next generation sequencing data and related statistics. The total mapped reads of each experimental group are shown. Additional details including the percentage of total mapped reads to exons, exon-exon junctions, and introns are indicated.
(XLSX)

S2 Table All genes identified in the *Drosophila* third-instar larval transcriptome and translatome. Genes in the transcriptome or translatome are defined as having at least 10 unique exon reads by transcriptional or ribosome profiling in all three replicates. Genes are listed in the order of transcription (transcriptional profiling RPKM). Genes uniquely found in only the transcriptome or translatome are listed at the end of each respective list.
(XLSX)

S3 Table Detailed analysis of the 100 genes determined to exhibit the lowest translation efficiency in wild type and Tor-OE. The unique ID term from Flybase (www.flybase.org) for each indicated gene is shown, along with the translation efficiency values (TE) in wild type and Tor-OE data sets. The additional columns contain RPKM value for each experimental replicate. Note that RpL3 is not among the list of genes with the lowest translation efficiency, but is included in this list for comparison with other ribosome subunits.
(XLSX)

S4 Table Detailed analysis of the 100 genes determined to exhibit the highest translation efficiency in wild type and Tor-OE. Flybase ID and gene symbol for each gene is shown, along with the translation efficiency (TE) values in wild type and Tor-OE data sets. The additional columns contain RPKM values for each experimental replicate.
(XLSX)

S5 Table Genes identified to exhibit significant change in transcription or translation efficiency in *GluRIIA* mutants compared to wild type using a reduced threshold of 1.5 fold change. Fold changes and p-values are presented for genes with significant adaptations in transcription or translation efficiency in *GluRIIA* versus wild type; 1.5 fold is used as a threshold. Transcriptional expression value and translation efficiency for each experimental replicate as well as the genomic location of the genes are also indicated.
(XLSX)

S6 Table Genes identified that exhibit altered transcription, translation, and increased translation efficiency in Tor-OE compared to wild type. List of genes with significantly increased translation efficiency in Tor-OE versus wild type are shown, along with fold changes and the associated significance (p-value) of translation efficiency. Fold change and associated p-value for ribosome profiling RPKM in Tor-OE versus wild type and *GluRIIA* versus wild type are also shown. Lists of genes with altered translation and altered transcription in Tor-OE versus wild type are also shown.
(XLSX)

S7 Table Genes significantly up-regulated in transcription or translation in Tor-OE compared to wild type. Significantly up-regulated genes are defined as having p-values of <0.05 (see Materials and Methods) and fold changes of >3 fold. Unique FlyBase ID terms and gene symbols are shown along with fold changes and associated p-values for ribosome and transcriptional profiling. P-values from two independent analysis tools are also indicated, using both the Baggerley’s test (CLC genomics work bench) and DESeq2 (R package).
(XLSX)
