# Supplementary figures and images for: Development of a tissue-specific ribosome profiling approach in Drosophila enables genome-wide evaluation of translational adaptations
Source: PLoS Genet. 2017 Dec 1;13(12):e1007117. doi: 10.1371/journal.pgen.1007117 (PMC5728580; doi:10.1371/journal.pgen.1007117)

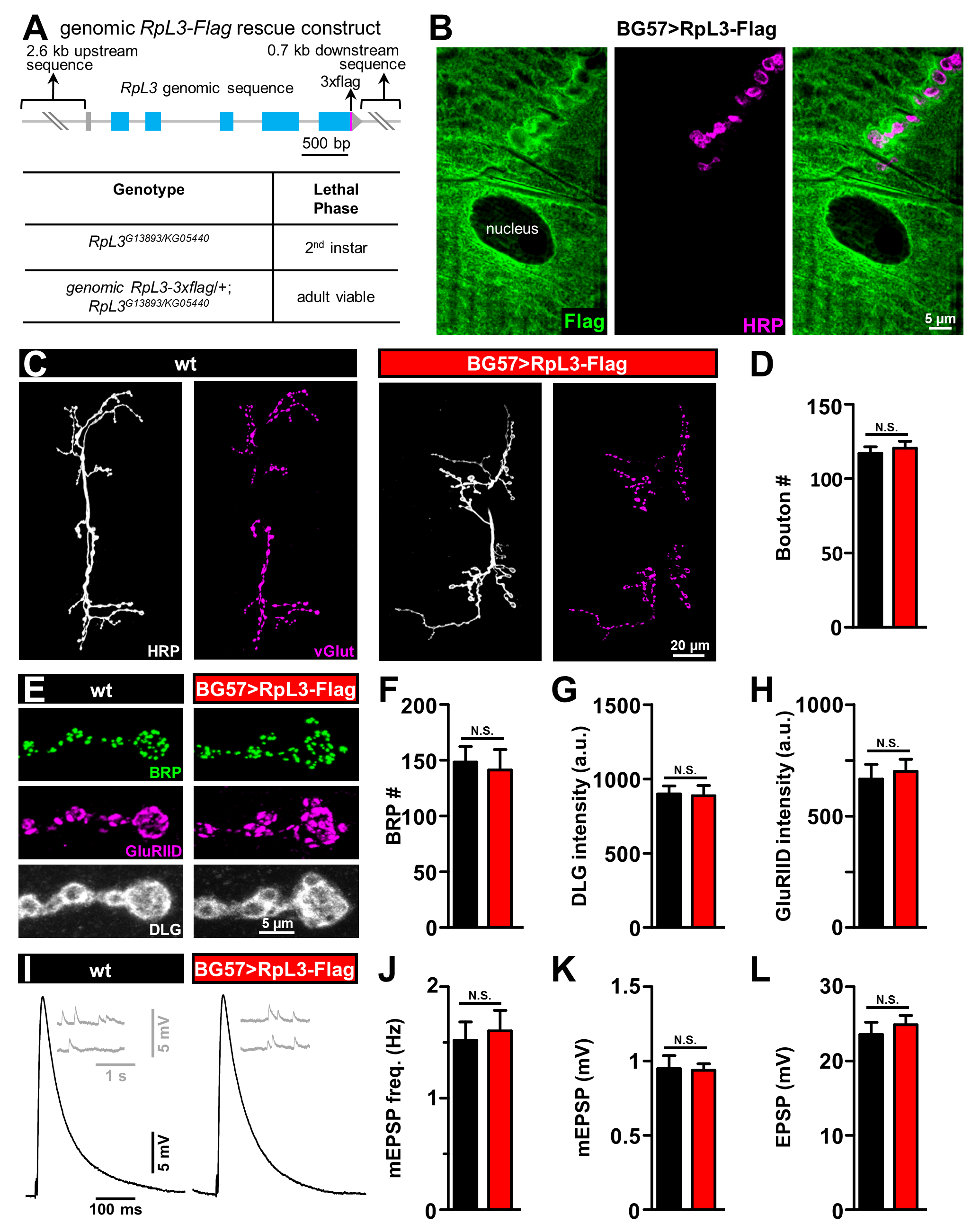

Supplement: S1 Fig — (A) Schematic illustrating the genomic RpL3-Flag rescue construct and table showing the lethal phase of RpL3 mutants (w;RpL3G13893/KG05440) compared to RpL3-Flag rescue (w;genomic-RpL3-3xflag/+; RpL3G13893/KG05440). (B) Representative images of larval muscles of BG57>RpL3-Flag (muscle overexpression of RpL3-Flag; w;BG57-Gal4/UAS-RpL3-3xflag) immunostained with anti-Flag (green) and anti-HRP (neuronal membrane marker; magenta) antibodies. (C) Representative images of muscle 6/7 NMJs from wild type (w1118) and BG57>RpL3-Flag immunostained with antibodies against vGlut (synaptic vesicle marker; magenta) and HRP (white). (D) Quantification of bouton number in the indicated genotypes. (E) Individual boutons of wild type and BG57>RpL3-Flag NMJs immunostained with antibodies against BRP (active zone marker; green), GluRIID (postsynaptic glutamate receptor marker; magenta), and DLG (postsynaptic density marker, white). No significant differences are observed in BRP number per NMJ (F), Dlg intensity (G), or GluRIID intensity (H) in wild type (n = 12) and BG57>RpL3-Flag (n = 12). (I) Representative traces of EPSP and miniature EPSP recordings from wild type and BG57>RpL3-Flag third-instar larval NMJs. No significant differences are observed in miniature EPSP frequency (J), miniature EPSP amplitude (K), or EPSP amplitude (L) in wild type (n = 11) and BG57>RpL3-Flag (n = 15). N.S = p>0.05; Student’s t test. (TIF) [file pgen.1007117.s001.tif]

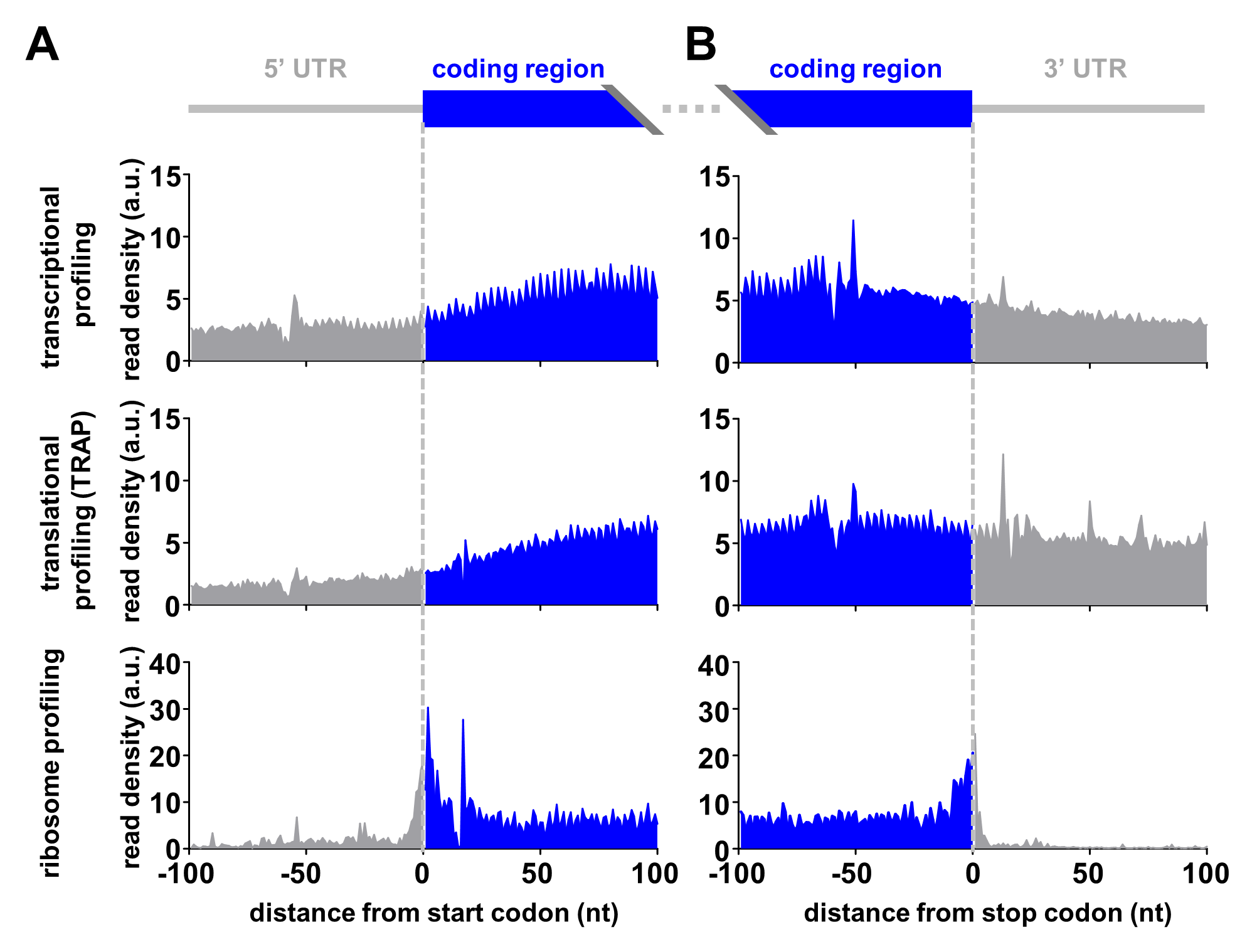

Supplement: S2 Fig — (A) Metagene analysis plot of averaged read density around the start codon for transcriptional, translational (TRAP) and ribosome profiling. (B) Metagene analysis plot of averaged read density around the stop codon for transcriptional, translational (TRAP) and ribosome profiling. Note the highly reduced density of ribosome profiling reads in the 3’UTR. UTR: untranslated region. (TIF) [file pgen.1007117.s002.tif]

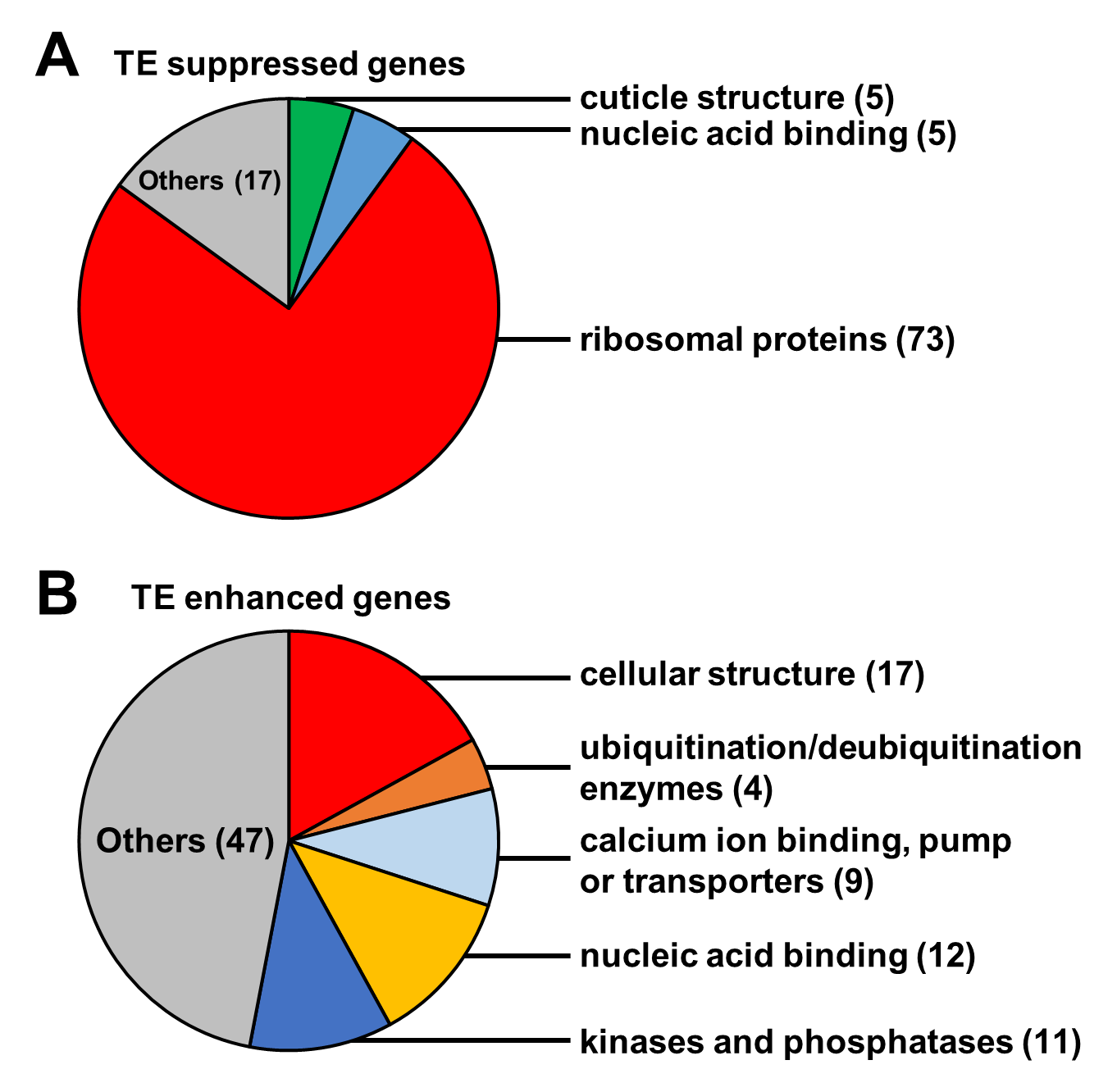

Supplement: S3 Fig — (A) Functional classes for the 100 genes with the lowest TE. Note that ribosomal proteins represent the largest class, with 73 of the 100 genes encoding ribosomal proteins. (B) Functional classes for the 100 genes with the highest TE. Diverse functional classes are present, with genes encoding proteins involved in cellular structure being the most abundant class. (TIF) [file pgen.1007117.s003.tif]

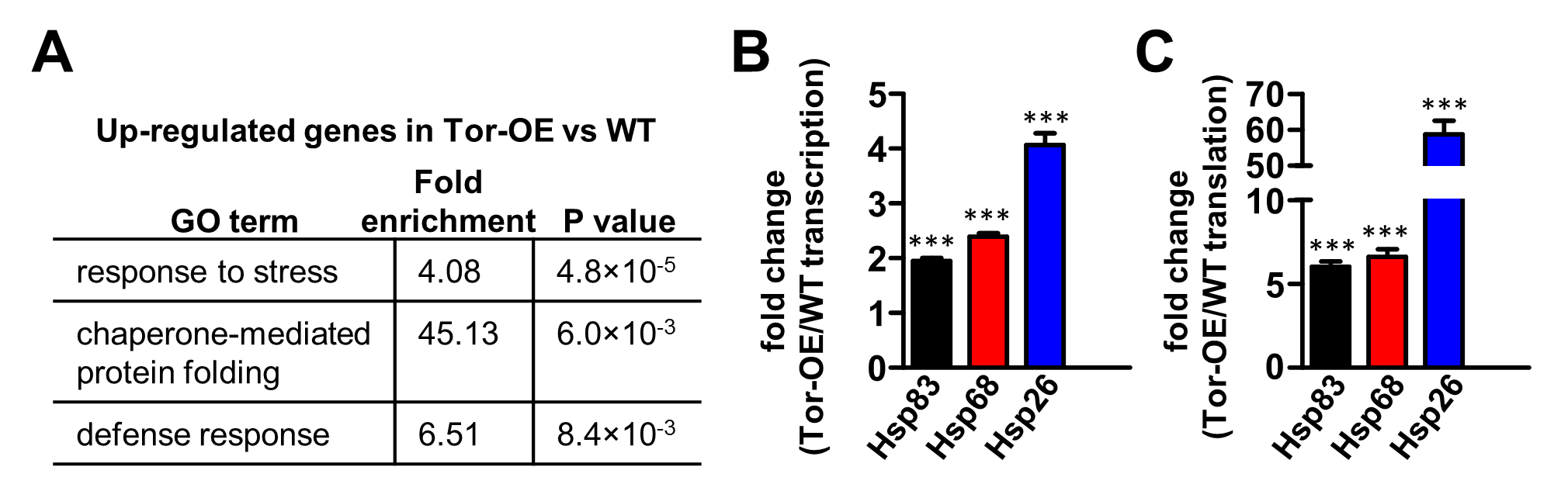

Supplement: S4 Fig — (A) GO term enrichment analysis of genes up-regulated in Tor-OE; a GO enrichment test is used to generate the p-values indicated. (B) Quantitative PCR (qPCR) analysis of three heat shock proteins (Hsp83, Hsp68, Hsp26) from wild type (w1118; BG57-Gal4/UAS-RpL3-3xflag) and Tor-OE (w1118;UAS-Tor-myc/+;BG57-Gal4/ UAS-RpL3-3xflag) larvae. Total RNA from preparations of each genotype was used as the template for the qPCR assay to assess transcriptional changes. (C) qPCR analysis of the indicated heat shock proteins and genotypes using ribosome associated RNA from the preparations as the qPCR template to assay translational changes. *** = p<0.001; Student’s t-test. (TIF) [file pgen.1007117.s004.tif]

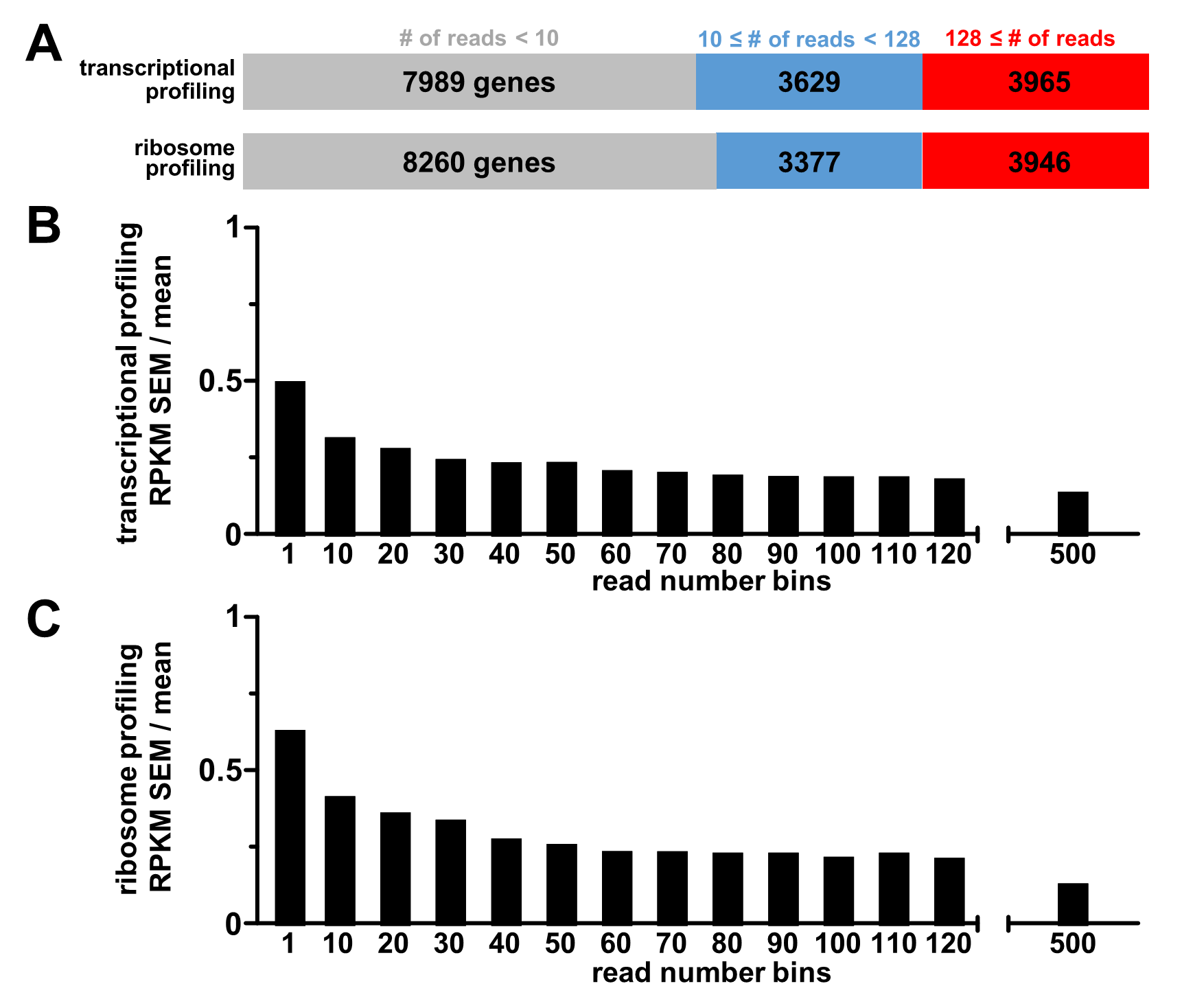

Supplement: S5 Fig — (A) Number of genes (of 15,583 total genes encoded in the Drosophila genome) that fall into each indicated read number range for transcriptional profiling and ribosome profiling. (B) Transcriptional profiling variability, defined as the standard error of the mean (SEM) normalized to the mean RPKM value of each gene, as a function of the number of mapped reads. Mapped reads for each gene were grouped and binned at indicated values. (C) The same plot as in B for reads obtained through ribosome profiling. Note that variability is sharply increased at read numbers below 10 in both transcriptional and ribosome profiling. (TIF) [file pgen.1007117.s005.tif]
